# Optogenetic Control of Non‐Apoptotic Cell Death

**DOI:** 10.1002/advs.202100424

**Published:** 2021-05-06

**Authors:** Lian He, Zixian Huang, Kai Huang, Rui Chen, Nhung T. Nguyen, Rui Wang, Xiaoli Cai, Zhiquan Huang, Stefan Siwko, Joel R. Walker, Gang Han, Yubin Zhou, Ji Jing

**Affiliations:** ^1^ Center for Translational Cancer Research Institute of Biosciences and Technology Texas A&M University Houston TX 77030 USA; ^2^ Department of Oral and Maxillofacial Surgery Sun Yat‐sen Memorial Hospital Sun Yat‐sen University Guangzhou Guangdong 510120 China; ^3^ Department of Biochemistry and Molecular Pharmacology University of Massachusetts Medical School Worcester MA 01605 USA; ^4^ Center for Epigenetics and Disease Prevention Institute of Biosciences and Technology Texas A&M University Houston TX 77030 USA; ^5^ Promega Biosciences LLC San Luis Obispo CA 93401 USA; ^6^ Center for Translational Cancer Research Institute of Biosciences and Technology Department of Translational Medical Sciences College of Medicine Texas A&M University Houston TX 77030 USA; ^7^ The Cancer Hospital of the University of Chinese Academy of Sciences (Zhejiang Cancer Hospital) Institute of Basic Medicine and Cancer (IBMC) Chinese Academy of Sciences Hangzhou Zhejiang 310022 China

**Keywords:** bioluminescence, necroptosis, optogenetics, pyroptosis, upconversion nanoparticles

## Abstract

Herein, a set of optogenetic tools (designated LiPOP) that enable photoswitchable necroptosis and pyroptosis in live cells with varying kinetics, is introduced. The LiPOP tools allow reconstruction of the key molecular steps involved in these two non‐apoptotic cell death pathways by harnessing the power of light. Further, the use of LiPOPs coupled with upconversion nanoparticles or bioluminescence is demonstrated to achieve wireless optogenetic or chemo‐optogenetic killing of cancer cells in multiple mouse tumor models. LiPOPs can trigger necroptotic and pyroptotic cell death in cultured prokaryotic or eukaryotic cells and in living animals, and set the stage for studying the role of non‐apoptotic cell death pathways during microbial infection and anti‐tumor immunity.

## Graphical abstract

A new class of genetically‐encoded actuators, designated LiPOPs, are designed to enable photoswitchable necroptosis or pyroptosis. LiPOPs are coupled with upconversion nanoparticles (UCNPs) or NanoLuc‐mediated luminescence‐aided optogenetic stimulation (NanoLOGS) to achieve wireless nano‐optogenetic or chemo‐optogenetic control of cell death *in vivo* with superior spatiotemporal precision. LiPOPs can also be exploited as safety switches to facilitate the development of safer cell‐based therapies.

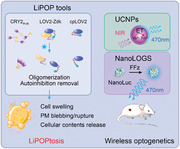

## 1 Introduction

Apoptosis, also known as non‐inflammatory programed cell death, is characterized by the activation of a series of cysteine‐aspartic proteases (caspases),^[^ ^1^ ^]^ and plays a critical role in the elimination of damaged cells to maintain tissue homeostasis.^[^ ^2^ ^]^ Necrosis is generally regarded as a form of uncontrolled cell death. However, recent studies have illuminated that necrosis is also tightly regulated under certain conditions, a process known as necroptosis.^[^ ^3^ ^]^ The necroptotic pathway can be initiated by death receptors, most often by tumor necrosis factor receptor 1 (TNFR1),^[^ ^4^ ^]^ followed by the successive activation of receptor‐interacting protein kinase 1 and 3 (RIPK1/3) and the mixed lineage kinase domain like (MLKL) protein.^[^ ^5^ ^]^ Activated MLKL undergoes oligomerization and migrates toward the plasma membrane (PM) to disrupt the membrane and cause the spillage of intracellular content into the surrounding tissues to induce inflammatory responses.^[^ ^6^ ^]^ Pyroptosis is another proinflammatory form of programmed cell death often initiated by extracellular or intracellular pathogen invasion.^[^ ^7^ ^]^ Unlike apoptosis, pyroptosis requires the concerted action of caspases 1, 3, 4, 5, or murine caspase 11.^[^ ^8^ ^]^ Upon inflammatory stimulation, one or more caspases are activated to form an inflammasome, and subsequently regulate the maturation and secretion of interleukin‐1 beta (IL‐1*β*) and interleukin‐18 (IL‐18).^[^ ^9^ ^]^ Similar to necroptosis and its specific effector molecule MLKL, pyroptosis is primarily executed by members of the gasdermin protein family (Gasdermin D, GSDMD and Gasdermin E, GSDME), which are cleaved by activated caspases to promote its self‐oligomerization and PM translocation, ultimately forming pores in the cell membrane to cause pyroptotic cell death.^[^ ^8^, ^10^ ^]^ Activation of necroptotic and pyroptotic cell death within the tumor microenvironment has been recently shown to enhance leukocyte‐mediated anti‐tumor immunity,^[^ ^11^ ^]^ thereby providing a unique cancer therapeutic opportunity.

Because key proteins involved in apoptosis, necroptosis, and pyroptosis can be initiated by the same caspases and sometimes share common upstream effectors, signaling cross‐talks among these cell death pathways are becoming increasingly appreciated in recent studies. For instance, necroptosis via TNFR1 can be disrupted by the apoptosis‐related Fas‐associated protein with death domain (FADD)‐caspase 8 complex. The activated FADD‐caspase 8 complex may disrupt necrosome formation by cleaving RIPK1 and destroying the RIPK3 oligomer to prevent the necroptotic process.^[^ ^12^ ^]^ Contrariwise, the blockade of caspase 8 by a caspase inhibitor Z‐VAD‐FMK can shift apoptosis to a necroptotic mode of cell death.^[^ ^13^ ^]^ Moreover, although apoptosis is highly controlled and immunologically silent, if apoptotic cells are not efficiently cleared by phagocytic cells, apoptosis can be accompanied by secondary necrotic or pyroptotic cell death, as characterized by passive cell swelling and immune responses.^[^ ^10b^ ^]^ Because these modes of cell death have frequent cross‐talk and overlap in some signaling steps, noninvasive tools capable of specifically committing cells to one cell death mode are highly needed for both research and therapeutic intervention purposes.

Optogenetics offers excellent opportunities for precise spatial and temporal control of physiological processes in live cells and tissues.^[^ ^14^ ^]^ We therefore take an optogenetic engineering approach to dissect essential steps involved in necroptotic and pyroptotic pathways by using light (LiPOPtosis), with the related tools designated light‐induced non‐apoptotic tools (LiPOPs). LiPOPs are generated by installing genetically‐encoded photosensory modules,^[^ ^15^ ^]^ derived from the Arabidopsis cryptochrome 2 (CRY2) or the Avena light‐oxygen‐voltage domain 2 (LOV2), into key proteins involved in necroptosis and pyroptosis. To overcome limited tissue penetration issues associated with blue light stimulation and minimize phototoxicity, we further couple LiPOP with upconversion nanoparticles (UCNPs) that permit near‐infrared (NIR)‐to‐blue emission, as well as bioluminescence catalyzed by NanoLuc, to achieve wireless control of cancer cell death in living animals. Finally, we apply these tools to precisely control the killing of bacteria and suicide of leukemia cells.

## 2 Results

### 2.1 Initiation of Necrosome Formation with Light

**Figure 1:**
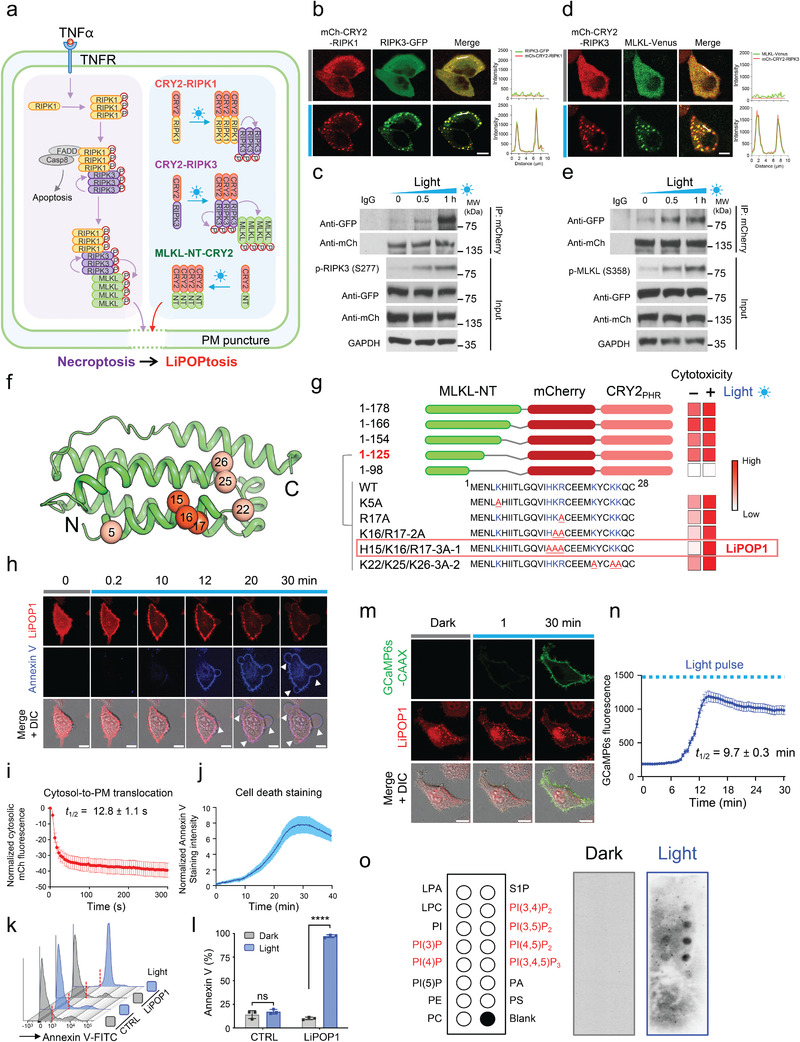
Optogenetic control of necrosome formation and necroptosis. Data were shown as mean ± s.e.m. Photostimulation was applied at 470 nm at a power density of 4 mW cm^−2^ or using a 488‐nm confocal laser (5% output). DIC, differential interference contrast. Scale bar, 10 µm. a) Schematics illustrating tumor necrosis factor alpha (TNFα)‐induced necroptosis under physiological conditions (left) and the design principle of converting necroptosis into LiPOPtosis (right), which enables light‐triggered necrosome formation and subsequent necroptotic cell death. The photo‐responsive region of cryptochrome 2 (CRY2_PHR_) is fused with RIPK1 or RIPK3 to manipulate necrosome formation with light. LiPOP1 is generated by fusing CRY2_PHR_ to an optimized N‐terminal fragment of MLKL (MLKL‐NT). In the dark, the engineeredCRY2‐MLKL‐NT exhibits minimal cytotoxic activity. Upon blue light illumination, CRY2 drives multimerization of MLKL‐NT to perforate PM to induce cell death, thereby mimicking RIPK1/3 induced phosphorylation of MLKL to expose the multimerizable NT domain to trigger necroptosis. NT, the N‐terminal domain of MLKL; PM, plasma membrane; RIPK1/3, receptor‐interacting serine/threonine‐protein kinase 1/3; FADD, Fas‐associated protein with death domain; Casp 8, caspase 8; P, phosphorylation site. b) Confocal images of HeLa cells co‐expressing mCh‐CRY2‐RIPK1 (red) and RIPK3‐GFP (green) before and after photostimulation. The intensity profiles of RIPK3‐GFP (green) and mCh‐CRY2‐RIPK1 (red; across the white line) in response to photostimulation were plotted on the right. Also see Movie S1, Supporting Information. c) Immunoblot analysis of light‐inducible association of RIPK1 with RIPK3 and phosphorylation of RIPK3. HeLa cells were co‐transfected with RIPK3‐GFP and mCh‐CRY2‐RIPK1 and subjected to photostimulation. Anti‐full length GFP (1:1000) and pRIPK3‐S277 antibodies (1:1000) were used to probe total RIPK3 and phosphorylated RIPK3, respectively. The duration of light stimulation was indicated above the blots. d) Confocal images of HeLa cells co‐expressing mCh‐CRY2‐RIPK3 (red) and MLKL‐Venus (green) before and after photostimulation. The intensity profiles of mCh‐CRY2‐RIPK3 and MLKL‐Venus (across the white line) in response to blue light were plotted on the right. Also see Movie S2, Supporting Information. e) Immunoblot analysis of the photo‐triggered RIPK3‐MLKL interaction and activation of MLKL reported by its phosphorylation. HeLa cells were co‐transfected with MLKL‐Venus and mCherry‐CRY2‐RIPK3 and subjected to photostimulation. Anti‐full length GFP (1:1000) and pMLKL‐S358 antibodies (1:1000) were used to probe total exogenous MLKL and its phosphorylation, respectively. f) The 3D structure of MLKL‐NT (PDB entry: 2MSV). Positively‐charged residues mutated in the study were indicated. g) Cartoon showing the design of MLKL‐NT‐mCherry‐CRY2_PHR_ hybrid constructs (termed as LiPOP). The cytotoxicity of each LiPOP variant before and after light illumination was summarized on the right. The triple‐mutation variant (H15A/K16A/R17A‐3A‐1; named as LiPOP1) showed the least dark activity while retaining a high degree of PM permeabilization upon photostimulation. The scale bar (white‐to‐red) indicates the relative degree of LiPOP‐mediated cytotoxicity. Also see Figure S2, Supporting Information. h–j) Time‐lapse confocal imaging of HeLa cells expressing LiPOP1 (red) upon exposure to blue light (h). Pacific Blue Annexin V (blue) was used as a PM marker and also as an indicator for PS translocation from the inner half leaflet of PM to the outer membrane during necroptotic cell death. Arrowheads indicate necroptotic bubble formation. Normalized fluorescence intensities of the cytosolic signal of LiPOP1 ((i), *n* = 62 cells from three independent assays) and Pacific Blue Annexin V staining ((j), *n* = 32 cells from three independent assays) upon photostimulation were also shown. Also see Movie S3, Supporting Information. k,l) light‐induced necroptotic cell death assessed by flow cytometry. HeLa cells expressing LiPOP1 were kept in the dark or exposed to blue light. Annexin V‐FITC was used to stain dying cells. HeLa cells expressing mCh‐CRY2 were used as CTRL. *n* = 3 (mean ± s.d.), **** *P* < 0.0001; ns, not significant (two‐tailed Student's *t*‐test). m,n) Time‐lapse confocal imaging of HeLa cells co‐expressing LiPOP1 (red) and GCaMP6s‐CAAX (green; (m)). The intracellular Ca^2+^ signals were reported by PM‐tethered GCaMP6s (*n* = 27 cells from three independent assays; (n)) Cells were subjected to pulsed light stimulation (1 s ON for every 30 s). Also see Movie S4, Supporting Information. o) A lipid strip assay to confirm the light‐dependent interaction between LiPOP1 and various phospholipids spotted on a nitrocellulose membrane. The exact layout of lipids on the membrane was shown on the left. LiPOP1‐expressing HEK293T cells were lysed and incubated with the lipid membrane with or without blue light illumination (right). An anti‐mCherry (1:2000) antibody was used to probe the lipid‐bound fraction of LiPOP1.

Upon death receptor activation (e.g., TNFR), necroptosis is initiated by the formation of a necrosome with RIPK1 and RIPK3 as two essential components. Oligomerized RIPK1 forms a platform to recruit RIPK3 and induce complex formation, with consequent auto‐phosphorylation of RIPK3. Activated RIPK3 further phosphorylates the downstream pseudokinase MLKL at residues threonine 357 and serine 358 (T357‐p/S358‐p). These posttranslational modifications lead to the exposure of the N‐terminal four helical bundle domain (4HBD) of MLKL (MLKL‐NT) to cause PM rupture and necroptosis (**Figure** **1**a).^[^ ^5^ ^]^ We first set out to reconstruct the necroptotic pathway via photo‐modulation of necrosome formation. To achieve this, we fused RIPK1 to an optical multimerizer, the N‐terminal photolyase‐homologous region of CRY2 (CRY2_PHR_),^[^ ^14b^ ^]^ which contains a flavin adenine dinucleotide (FAD) cofactor and undergoes monomer‐to‐oligomer transition upon blue light illumination (Figure 1a). We used a human cervical cancer cell line, HeLa, as an ideal reconstitution system since it lacks endogenous expression of RIPK3.^[^ ^16^ ^]^ In the dark, both mCherry (mCh)‐CRY2‐RIPK1 and RIPK3‐GFP exhibited an even distribution in the cytosol of transfected HeLa cells. Upon brief light stimulation at 470 nm, we noted a rapid oligomerization of mCh‐CRY2‐RIPK1 and the co‐clustering of RIPK3‐GFP (Figure 1b; Figure S1a and Movie S1, Supporting Information; *t*_1/2_, _on_ = 3.6 ± 0.7 min), suggesting an interaction between RIPK1 and RIPK3. We further independently confirmed the light‐dependent interaction by co‐immunoprecipitation (Co‐IP). Upon exposure to blue light for 0, 0.5, or 1 h, we noted a gradual increase in RIPK3‐GFP immunoprecipitated by RIPK1, along with escalating phosphorylation of RIPK3 at serine 277 (Figure 1c). Under normal physiological conditions, tumor necrosis factor‐*α* (TNF*α*) /TNFR1‐initiated necroptosis, also cross‐talks with key signaling molecules involved in apoptotic cell death, including FADD and caspase 8, adding complications to assigning the exact roles of RIPK and MLKL during cell death signaling. Caspase 8 is recruited by oligomerized RIPK1, and cleaves RIPK1 and RIPK3 to shift necroptosis to apoptosis. To overcome this issue, we sought to directly trigger RIPK3 oligomerization using light but without the involvement of upstream RIPK1. We hence extended a similar engineering approach to RIPK3, aiming to validate whether RIPK3 oligomerization is the upstream cause for MLKL activation and necroptotic initiation.^[^ ^17^ ^]^ As anticipated, we observed light‐inducible formation of mCh‐CRY2‐RIPK3 puncta, followed by co‐clustering of MLKL‐Venus (Figure 1d; Figure S1b and Movie S2, Supporting Information) and increased phosphorylation of MLKL upon photo‐illumination (Figure 1e), a clear indication of MLKL activation. In parallel, we recapitulated similar phenotypes in human 786‐O renal carcinoma cells (Figure S1c–f, Supporting Information) and rodent B16 melanoma cells (Figure S1g–j, Supporting Information). Collectively, by harnessing the power of light while bypassing death receptor activation, we have provided compelling evidence to support a model in which the initial steps of necroptosis follow the order of RIPK1→RIPK3→MLKL in various mammalian cell lines.

### 2.2 Optogenetic Mimicry of MLKL‐Mediated Necroptosis

The N‐terminal domain of MLKL, particularly the 4HBD region (Figure 1f), is directly involved in the execution of necroptosis by triggering a series of intracellular events, including i) self‐oligomerization and PM translocation, ii) the exposure of PM‐resident phosphatidylserine (PS) toward the extracellular space, and iii) Ca^2+^ influx.^[^ ^6^ ^]^ To recapitulate these essential steps during necroptosis, we set out to fuse varying fragments of MLKL‐NT with mCh‐CRY2 (Figure 1g). We envisioned that light‐induced oligomerization of MLKL‐NT could elicit similar phenotypes (Figure 1a). By using Annexin V staining of externally‐exposed PS as a readout for cell death, we found that most tested MLKL‐NT fragments (1–178, 1–166, 1–154, and 1–125) exhibited a high basal cytotoxic activity even in the absence of light stimulation (Figure 1g; Figure S2a, Supporting Information), indicating the high potency of these fragments to perforate the PM. Further truncation to residue 98 abolished the PM‐disrupting ability of MLKL‐NT, suggesting that the structural integrity of the full 4HBD is required for the membrane‐perforating activity.

**Figure 2:**
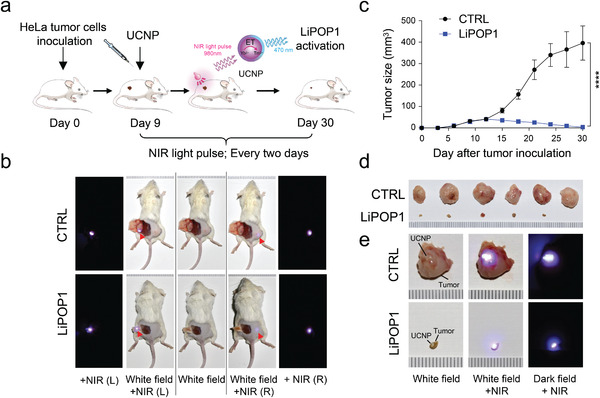
Nano‐optogenetic control of tumor cell necroptosis *in vivo*. Data were shown as mean ± s.d. a) Schematics illustrating the *in vivo* experimental setup. 150 µg of UCNPs were injected into each tumor site of SCID‐beige mice 9 days after inoculation of 1 × 10^6^ LiPOP1‐expressing HeLa cells. Mice were subjected to pulsed NIR light stimulation (980 nm at a power density of 250 mW cm^−2^; 20 s ON + 5 min OFF; 2h every three days). At day 30, mice were euthanized for tumor isolation and phenotypic analyses. b) Visualizing UCNPs after subcutaneous injection into the tumor sites. Images were acquired in the same mouse under three conditions: white field without NIR light (middle); white field with NIR light at the surgical exposure site, or in the dark room with NIR light (left); and white field with NIR light at the injection site, or in the dark room with NIR light (right). Both UCNPs in the surgically exposed sites (left) and subcutaneously buried sites (right) showed prominent blue light emission upon NIR stimulation, attesting to the superior tissue penetration of NIR light. c,d) Tumor sizes at the indicated days were measured by a digital caliper with the tumor volume calculated in mm^3^ ((length × width^2^)/2). **** *P* < 0.0001 compared to the control groups (two‐tailed Student's *t*‐test; *n* = 6; mean ± s.d.). Images of isolated tumor xenografts for each group were shown in panel (d). e) Representative images of UCNP‐containing tumors for the control or LiPOP1 groups with (middle) or without NIR light excitation in the white field (left) or dark room (right).

To reduce the baseline (dark) activation of MLKL‐NT fragments, we reasoned that neutralization of positively‐charged lysine or arginine residues might reduce their PM‐perforating function. Indeed, the introduction of single (R17A), double (2A; K16A/R17A), or triple mutations substantially reduced the basal cytotoxicity of MLKL‐NT (residues 1–125, Figure 1g; Figure S2a, Supporting Information). Among the two tested triple mutants (3A1 and 3A2), the combination of H15A/K16A/R17A showed the least basal activity but retained its PM‐puncturing activity upon light illumination, hence named as LiPOP1 for light‐induced non‐apoptotic (LiPOPtotic) tool 1 (Figure 1g). In HeLa cells, we observed light‐dependent translocation of LiPOP1 from the cytosol to the PM with a half‐life of 12.8 ± 1.1 s (Figure 1h;i; Movie S3, Supporting Information), accompanied by a gradual appearance of Annexin V staining in the PM due to PS exposure (Figure 1j; *t*_1/2_ = 18.0 ± 4.0 min). We found that LiPOP1 led to the killing of almost all the cells within 30 min of pulsed blue light illumination. As a stringent control, no overt phototoxic side effects were observed for the same cells expressing mCh‐CRY2 (Figure 1k,l). In the control group, HeLa cells expressing MLKL‐NT 3A (H15A/K16A/R17A)‐mCh did not show signs of cell death under the same photostimulation condition (FigureS2b, Supporting Information). In parallel, in HeLa cells co‐transfected with LiPOP1 and a genetically‐encoded Ca^2+^ indictor GCaMP6m, we noted pronounced Ca^2+^ influx due to PM disruption upon photostimulation (Figure 1m; Movie S4, Supporting Information), with the half‐life determined to be 9.7 ± 0.3 min (Figure 1n).

Next, we explored the mechanistic basis of LiPOP1 by asking whether it could physically interact with negatively‐charged PM‐resident phosphoinositides (PIPs) as activated MLKL does.^[^ ^6^ ^]^ When shielded from blue light, LiPOP1 did not show appreciable interaction with phospholipid species immobilized on a nitrocellulose membrane (Figure 1o). Under blue light stimulation, LiPOP1 bound most strongly to PI(3,5)P_2_, PI(4,5)P_2_, and PI(3,4,5)P_3_ (species that are most abundant in the PM), but less strongly to PI(3, 4)P_2_,PI3P, and PI4P species that are more abundant in subcellular organelles (Figure 1o).

Collectively, LiPOP1 retains the MLKL PIP‐binding capability to engage and perforate membranes, which allows us to photo‐trigger necroptosis by bypassing conventional death receptor activation. With LiPOP1, we can achieve both photo‐tunable control of Ca^2+^ influx and necroptotic cell death by stimulation with brief light pulses.

### 2.3 Nano–Optogenetic Control of Tumor Necroptosis *In Vivo*

To validate *in vivo* the light‐induced cell killing mediated by LiPOP1, we generated a xenograft mouse model of solid tumors by injecting HeLa‐LiPOP1 cells or HeLa‐mCh‐CRY2 as the negative control into the flanks of immunodeficiency mice (**Figure** **2**). Visible light in the blue–green emission range typically can penetrate human skin to a depth of less than 2–3 mm,^[^ ^18^ ^]^ which significantly limits the application of optogenetic tools *in vivo*. To enable wireless optogenetic intervention *in vivo*, we further combined LiPOP1 with upconversion nanoparticles (UCNPs) that have near infrared (NIR)‐to‐blue upconversion luminescence capability. The UCNPs act as nano‐transducers that allow inducible activation of LiPOP1 in living animals by converting deep tissue‐penetrable NIR light into blue light emission. In order to match the absorption window of CRY2, we used previously‐developed mono‐dispersed 40‐nm *β*‐NaYF_4_: Yb, Tm@β‐NaYF_4_ UCNPs that exhibit bright blue emission upon 980 nm irradiation^[^ ^19^ ^]^ (Figure 2a, insert panel). After 9 days of tumor establishment, the tumor sites were injected with UCNPs and subjected to pulsed NIR light stimulation (980 nm at a power density of 250 mW cm^−2^; 2 h for every three days; pulses of 20 s ON + 5 min OFF). We detected bright blue light emission from the injection sites following exposure to a brief pulse of NIR light illumination (Figure 2b). At day 30 after tumor establishment, the HeLa‐LiPOP1 group showed a substantial reduction in tumor size compared with the control group (HeLa expressing mCh‐CRY2) upon NIR light stimulation (Figure 2c–e). Together, these findings establish that our NIR light‐tunable nano‐optogenetic platform could enable wireless optical control of LiPOP1‐mediated necroptosis to facilitate tumor killing *in vivo*.

### 2.4 Chemo–Optogenetic Control of Tumor Cell Necroptosis *In Vitro* and *In Vivo*

UCNPs have spatiotemporal precision as NIR light could be withdrawn anytime, and have an improved penetration depth (up to 2–3 cm).^[^ ^19^ ^]^ However, while effective for moderate tissue depths *in vivo*, this strategy will likely remain less effective for tissues deeply buried within the body. To overcome this roadblock and enable more extensive applications *in vivo*, we explored another biocompatible approach to photoactivate LiPOP1. NanoLuc, an ATP‐independent luciferase, offers high intensity, glow‐type luminescence by catalyzing the conversion of the substrate furimazine (Fz) into furimamide, with photons emitting at 460 nm.^[^ ^20^ ^]^ The emission overlaps with the ideal photoactivation window (450–470 nm) of blue light‐responsive optogenetic devices. NanoLuc has been successfully used to generate bioluminescence and subsequently photo‐activate a blue–green optogenetic system via bioluminescence resonance energy transfer (BRET).^[^ ^21^ ^]^ However, its potential to directly activate CRY2‐based optogenetic tools with temporal precision *in vivo* is yet to be explored.

**Figure 3:**
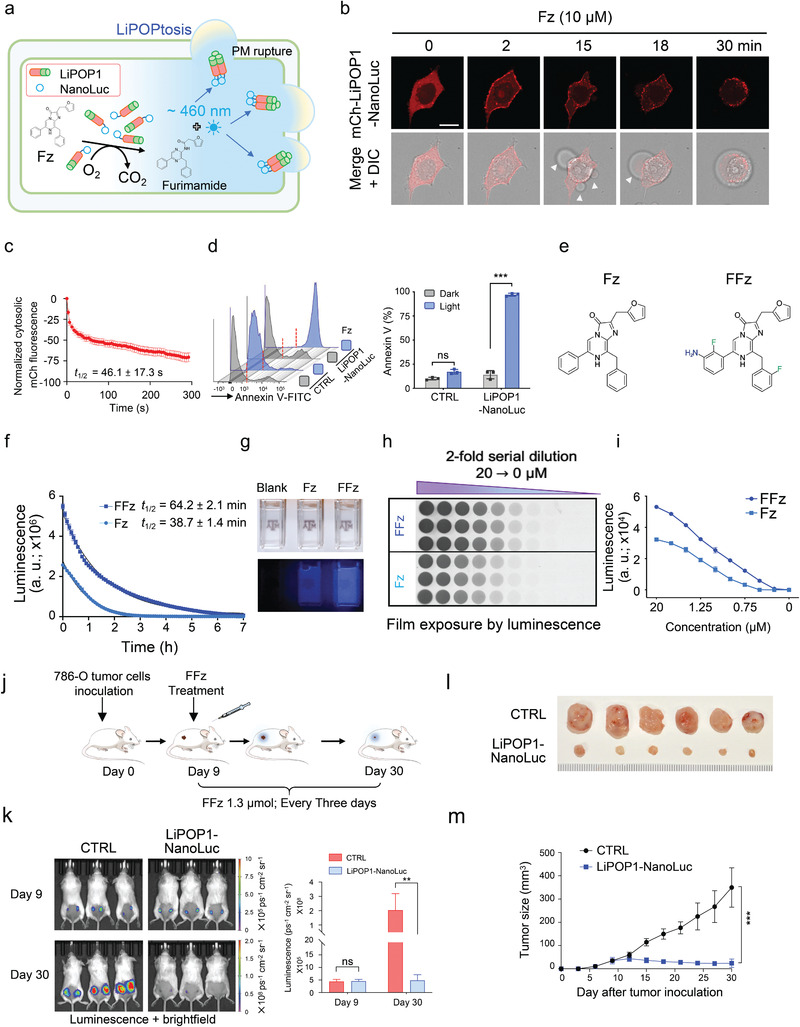
Chemo‐optogenetic control of tumor necroptosis with NanoLOGS. a) Fusion of NanoLuc to LiPOP1 enables luminescence‐aided optogenetic stimulation (NanoLOGS). NanoLuc catalyzes the conversion of furimazine (Fz) into furimamide with concomitant release of photons that emit at 460 nm to photoactivate the CRY2 photoreceptors, thereby causing oligomerization of LiPOP1 to perforate the PM and elicit necroptosis. b) Time‐lapse confocal imaging of 786‐O tumor cells with stable expression of LiPOP1‐NanoLuc upon treatment with 10 µm Fz. White arrowheads indicated necroptotic bubble formation. DIC, differential interference contrast. Also see Movie S5, Supporting Information. c) Quantification of cytosolic mCherry signals from mCh‐LiPOP1‐NanoLuc in 786‐O cells following addition of 10 µm Fz (*n* = 57 cells from three independent assays; mean ± s.e.m.). d) Optochemical induction of necroptotic cell death assessed by flow cytometry. 786‐O cells expressing LiPOP1‐Nanoluc treated with or without 10 µm Fz for 30 min. Annexin V‐FITC was used to stain dying cells. 786‐O cells expressing mCh‐NanoLuc‐CRY2 were used as CTRL. *n* = 3 (mean ± s.d.). *** *P* < 0.001 compared to the CTRL; ns, not significant (two‐tailed Student's *t*‐test). e) Chemical structures of Fz and its derivative fluorofurimazine (FFz), the latter of which has improved photochemical properties and biosafety profiles. f) Luminescence decay in 786‐O cells expressed mCh‐NanoLuc‐CRY2 after treatment with 10 µm Fz or FFz. The half‐lives (*t*_1/2_) were determined to be 38.7 ± 1.4 min (Fz) and 64.2 ± 2.1 min (FFz), respectively. g) Real‐time visualization of bioluminescence in 786‐O cells. Tumor cells stably expressing mCh‐NanoLuc‐CRY2 were detached and resuspended in phenol‐free cell culture media. The emission from the cell suspension upon 10 µm Fz or 10 µm FFz addition was recorded. h,i) A dot blot assay showing luminescence intensities of 786‐O cells expressing mCh‐NanoLuc‐CRY2 at 5 min after Fz or FFz treatment. An X‐ray film was covered above a 96‐well plate containing transfected 786‐O cells to detect the luminescence in a dark room. Quantification of the results was shown in panel (i). *n* = 3 independent biological replicates. j) Cartoon showing the experimental design for *in vivo* experiments. Scid‐beige mice were subcutaneously inoculated with 786‐O tumor cells expressing mCh‐NanoLuc‐CRY2 (CTRL) or LiPOP1‐NanoLuc, and 1.3 µmol FFz was injected every three days starting from day 9 till day 30 for chemo‐optogenetic activation of LiPOP1‐NanoLuc or its control. k) Bioluminescence imaging of scid‐beige mice engrafted with mCh‐Nanoluc‐CRY2 (CTRL) or LiPOP1‐NanoLuc‐expressing 786‐O tumor cells at day 9 and day 30. Right, mean bioluminescence signal intensity of tumors. ** *P* < 0.01; ns, not significant (two‐tailed Student's *t*‐test; *n* = 6 tumors; mean ± s.d.). l,m) Representative images of tumors isolated from scid‐beige mice bearing mCh‐Nanoluc‐CRY2 (CTRL) or LiPOP1‐NanoLuc‐expressing 786‐O xenografts with and without 1.3 µmol (for 25‐g mouse) FFz treatment (l). Tumor growth curve at the indicated time points was measured by a digital caliper with the tumor volume calculated in mm^3^ ((length × width^2^)/2). ((m), *n* = 6 tumors; *** *P* < 0.001 compared to the control group).

We hence sought to design a NanoLuc‐mediated luminescence‐aided optogenetic stimulation (NanoLOGS) platform to examine whether Fz as the substrate of NanoLuc could activate LiPOP1 via CRY2 oligomerization (**Figure** **3**a). We generated a hybrid protein by fusing NanoLuc with LiPOP1 (LiPOP1‐NanoLuc). To enable semi‐quantitative estimation of the strength of bioluminescence, we used a convenient dot blot‐like assay, in which the bioluminescence led to the exposure of an X‐ray film (Figure S3a, Supporting Information). In NanoLuc‐ or LiPOP1‐NanoLuc expressing cells cultured in a 96‐well plate after treatment with Fz, we found a dose‐dependent change in bioluminescence (Figure S3a,b, Supporting Information). The generated bioluminescence was strong enough to be visualized by the naked eye in a dark room (Figure S3c, Supporting Information). Confocal imaging further revealed that mCh‐NanoLuc‐CRY2 could be efficiently activated in the presence of 10 µm Fz, as reflected by oligomerization of the hybrid protein (Figure S3d–f, Supporting Information). We observed photo‐triggered PM translocation of LiPOP1‐NanoLuc and necroptosis in three cancer cell lines, including human HeLa cells, murine melanoma (B16) cells (Figure S3g, Supporting Information), and renal adenocarcinoma cells (786‐O, Figure 3b), in response to Fz treatment. In 786‐O cells expressing LiPOP1‐NanoLuc, we observed PM translocation (*t*_1/2_: 46.1 ± 17.3 s, Figure 3b,c) and necroptotic cell death within 30 min upon treatment with 10 µm Fz (Figure 3d; Movie S5, Supporting Information). The transfection of mCh‐NanoLuc‐CRY2 alone did not cause cell death within 30 min after Fz treatment (Figure S3e, Supporting Information), fully demonstrating the general applicability of LiPOP1‐NanoLuc for tumor killing *in vitro*. The half‐life of the NanoLOGS method was about four times as long as direct blue light stimulation (46.1 s vs 12.8 s. Figures 1i and 3c), likely due to the relatively lower power density of bioluminescence. However, the degree of mCherry translocation to the PM remained comparable between Fz treatment and blue light stimulation (Figures 1h,i and 3b,c) suggesting that the NanoLOGS approach is as effective as exogenous photostimulation.

The solubility and biosafety of substrate is critical for the application of the NanoLOGS platform *in vivo*. Recent studies have shown that the brightness of furimazine‐induced bioluminescence in mice is limited by its poor solubility and bioavailability.^[^ ^22^ ^]^ In addition, furimazine has been shown to cause liver toxicity after 7 days of injection at ≈0.052 µmol per 25g animal.^[^ ^23^ ^]^ A new substrate, fluorofurimazine (FFz,Figure 3e), was developed to overcome these problems.^[^ ^22^ ^]^ FFz has been shown to have higher brightness, improved aqueous solubility, and better safety profiles when administered into mice.^[^ ^22^ ^]^ Indeed, in our side‐by‐side comparison between Fz and FFz *ex vivo*, we found that the bioluminescence decay half‐life was prolonged almost by twofold (38.74 ± 1.43 min for Fz *vs* 64.2 ± 2.1 min for FFz; Figure 3f). In addition, based on the dot blot assay and imaging results, the bioluminescence brightness was enhanced by ≈1.5–1.8‐fold at the same concentration (Figure 3g–i). Hence, we used FFz to demonstrate the application of LiPOP1‐NanoLuc for tumor killing *in vivo*.

To examine the biocompatibility of FFz, we next moved on to characterize the potential *in vitro* cytotoxicity and *in vivo* biosafety of FFz. FFz did not exert appreciable cytotoxicity to mammalian cells up to 20 µm concentration (Figure S4a, Supporting Information). Furthermore, we performed histopathological and routine blood analyses on major organs and tissues obtained from mice treated with FFz (1.3 µmol for 25‐g mouse (as described previously)^[^ ^22^ ^]^) every three days (a total of 7 doses) for up to three weeks. PBS‐injected mice were used as controls. We found that neither the PBS nor the FFz treated groups displayed noticeable tissue damage or inflammatory lesions (Figure S4b,c, Supporting Information), Furthermore, in the complete blood count (CBC) test, most tested parameters fell within normal ranges (Table S1, Supporting Information). These results clearly suggest negligible toxicity of FFz to major organs, including heart, liver, spleen, lung, kidney, muscles, and the circulatory system. Clearly, we have established FFz as an improved substrate for the NanoLOGS platform, which has greater solubility, higher brightness, prolonged luminescence, and good biocompatibility *in vivo*.

Having validated the superior performance of FFz, we moved on to test the photochemical properties of NanoLOGS in vivo. We subcutaneously inoculated engineered human 786‐O cells into immunodeficient mice, with dorsal flanks receiving 786‐O cells expressing either LiPOP1‐NanoLuc or the control, mCh‐NanoLuc‐CRY2 (Figure 3j). After tumor establishment for 9 days, we performed intratumoral injection of FFz (1.3 µmol for 25‐g mouse) every 3 days for seven times. At the endpoint (day 30), the LiPOP1‐NanoLuc group showed a significant reduction in the tumor size compared to the control group (Figure 3k–m). Collectively, these findings establish NanoLOGS as a safe wireless method to effectively activate CRY2‐based optogenetic devices to kill tumor cells both *in cellulo* and *in vivo*.

### 2.5 Optogenetic Control of GSDMD‐Mediated Pyroptosis

**Figure 4:**
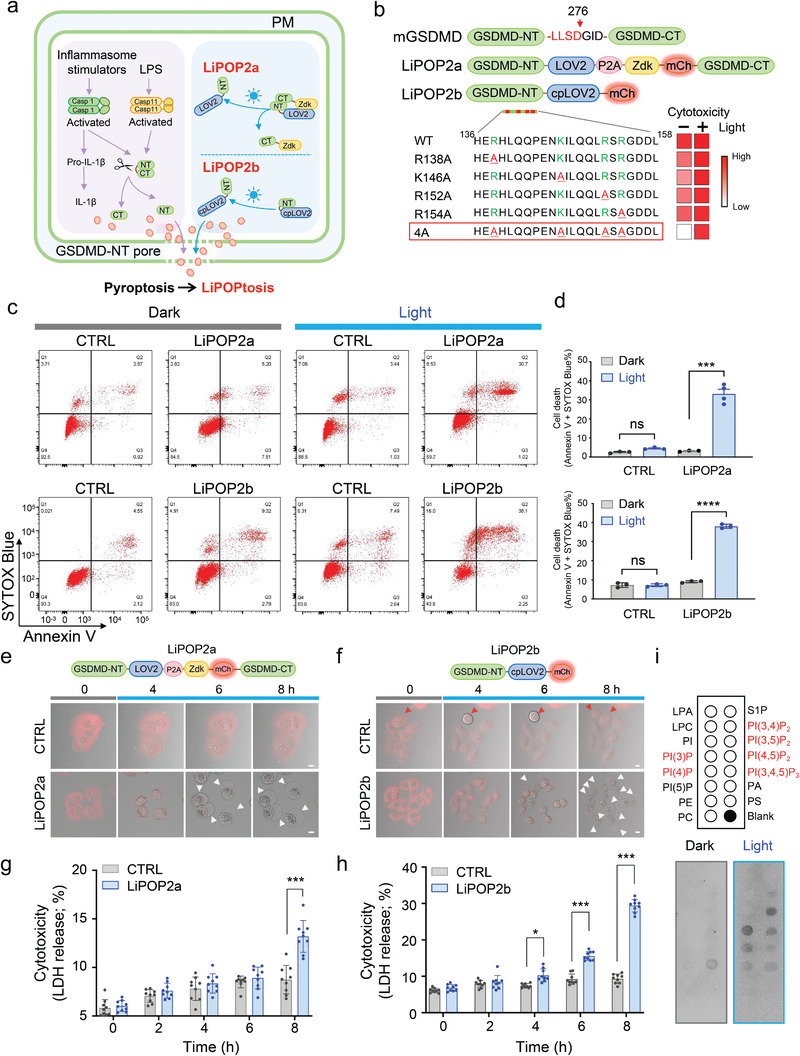
Optogenetic control of pyroptosis. Photostimulation was applied at 470 nm at a power density of 4 mW cm^−2^ or using a 488‐nm confocal laser (5% output). ns, not significant; * *P* < 0.05; ***P < 0.001; ****P < 0.0001 when compared to the corresponding control group (two‐tailed Student's *t*‐test). Scale bar, 10 µm. a) Schematic depiction of pyroptotic induction (left) and the design of LiPOP2a and LiPOP2b (right). Ligand or stress‐induced activation of caspase 1 and caspase 11 leads to the cleavage of GSDMD, thereby releasing the N‐terminal pore‐forming fragment of GSDMD (GSDMD‐NT) to cause pyroptosis. In LiPOP2a, GSDMD‐NT is brought into proximity with GSDMD‐CT via the LOV2‐Zdk association in the dark. Upon light stimulation, Zdk dissociates from LOV2 to expose the cytotoxic GSDMD‐NT fragment to induce pyroptosis. For the design of LiPOP2b, cpLOV2 is fused downstream of an engineered GSDMD‐NT fragment, which imposes steric hindrance upon GSDMD‐NT to prevent PM perforation. Upon photostimulation, the unfolding of the Ja helix in cpLOV2 causes the exposure of GSDMD‐NT to restore its pore‐forming capability. b) Domain architectures of LiPOP2 hybrid constructs. The cytotoxicity of each construct before and after light illumination was summarized. The hybrid variant (R138A/K146A/R152A/R154A; named as LiPOP2b) highlighted in the red box showed the least dark activity but restored its cytotoxic activity upon blue light illumination. Heat map (white‐to‐red) indicates the degree of cytotoxicity. c) Cell viability assessed by Annexin V and SYTOX Blue staining. Transfected HeLa cells were either shielded or exposed to blue light for 6 h, and then subjected to flow cytometry analysis. d) Quantification of double positive HeLa cells expressing LiPOP2a (top) or LiPOP2b (bottom). Data were shown as mean ± s.d. from three independent assays. e,f) Time‐lapse confocal imaging of HeLa cells stably expressing LiPOP2a (e) or LiPOP2b (f) following blue light stimulation. CTRL, the control groups (LOV2‐P2A‐Zdk‐mCh‐GSDMD‐CT for LiPOP2a; cpLOV2‐mCh for LiPOP2b). White arrowheads indicated pyroptotic bubble formation. One mitotic cell was observed in panel (f, red arrowhead), indicating that LiPOP2 expression did not perturb cell division. Also see Movies S6 and S7, Supporting Information. g,h) Quantification of LDH release from HeLa cells expressing LiPOP2 or the corresponding control groups (the same as described in (e,f) at the indicated time‐points following photostimulation. Data are represented as mean ±s.d. from nine independent assays. i) Light‐dependent association between LiPOP2b and the indicated lipids spotted on a nitrocellulose membrane. Purified MBP‐tagged LiPOP2b was incubated with the lipid membrane with or without blue light illumination. An anti‐MBP‐antibody was used to probe the bound fraction of LiPOP2b.

We next applied our optogenetic engineering approach to reconstruct the key steps involved in pyroptosis, a programmed cell death pathway initiated by inflammatory caspase activation and involving the gasdermin protein family.^[^ ^24^ ^]^ As the initiator caspase, human caspase‐1 / caspase‐4 (or murine caspase‐11) exists as an inactive monomer and requires dimerization or oligomerization, often triggered by pathogen‐ or host‐derived danger signals, to induce pyroptosis.^[^ ^9^, ^25^ ^]^ Upon autoactivation via oligomerization, human caspase‐1 or mouse caspase‐11 is able to cleave its substrate gasdermin D (GSDMD) in the linker region to release intramolecular autoinhibition and generate a 30‐kDa N terminal fragment (GSDMD‐NT or p30) fragment, which can effectively form pores in the PM (**Figure** **4**a, left).^[^ ^10a^ ^]^

To achieve optical control of GSDMD cleavage and/or cytotoxic GSDMD‐NT release, we employed two different engineering approaches, including light‐inducible GSDMD autoinhibition mimicry, and conditional uncaging of GSDMD‐NT (Figure 4a, right). First, to mimic the release of intramolecular autoinhibition of GSDMD between its N‐ (GSDMD‐NT: pore‐forming domain) and C‐terminal regions (GSDMD‐CT: repressive domain),^[^ ^24^ ^]^ we took advantage of a light‐inducible dissociation system, LOV2 Trap and Release of Protein (LOVTRAP), in which an affibody Zdark (Zdk) interacts with LOV2 in the dark but dissociates rapidly upon photostimulation.^[^ ^26^ ^]^ We fused the GSDMD‐NT and GSDMD‐CT fragments with LOV2 and Zdk, respectively, and co‐expressed them at a near 1:1 ratio using a bicistronic expression vector based on the P2A self‐cleavage peptide (LiPOP2a; Figure 4b). We reasoned that the light‐induced dissociation between GSDMD‐NT and GSDMD‐CT could overcome the intramolecular autoinhibition as caspase 11 does by cleaving the middle linker between residues D276 and G277 (Figure 4b).^[^ ^27^ ^]^ In the dark, HeLa cells expressing the construct showed minimal cell death, with around 5% of cells exhibiting a double‐positive staining for the cell death markers SYTOX Blue and Annexin V (Figure 4c, top; Figure S5a,b, Supporting Information). Following light stimulation, we noted a pronounced increase in cell death, with the double‐positive populations soaring to about 30% (Figure 4d, top). Time‐lapse confocal imaging further confirmed the flow cytometry results. In LiPOP2a‐expressing cells, we observed light‐inducible pyroptotic cell death within 8 h, while the control group showed no significant increase in cell death under the same conditions (Figure 4e; Movie S6, Supporting Information). In another independent assay, we monitored cytotoxicity by measuring cell culture medium activity of lactate dehydrogenase (LDH), a cytosolic enzyme that is released upon damage to the PM.^[^ ^28^ ^]^ We noted a time‐dependent increase in LDH release in the LiPOP2a group but not in the control group (Figure 4g). Taken together, these findings validate that the GSDMD‐CT domain exerts a potent autoinhibitory effect on the cytotoxic GSDMD‐NT domain.

In a second approach, we took one step further by fusing the toxic domain of GSDMD (GSDMD‐NT) with a photoswitch modified from LOV2 to generate a more compact version. The fusion of protein tags at the N‐terminus of GSDMD tends to compromise its pore‐forming function. We therefore slightly modified the photo‐responsive system by using a circularly permuted LOV2 (cpLOV2), in which the original N/C‐termini were covalently connected by a linker while creating new N/C‐termini at residues G516 and T517 (Figure S6a–c, Supporting Information). This new photoswitchable module allows us to make a hybrid construct by leaving the GSDMD‐NT unmodified while retaining the photosensitivity (Figure 4b; Figure S5c, Supporting Information). We hypothesized that the cpLOV2 would exert steric hindrance toward GSDMD‐NT, thereby prohibiting its pore‐forming activity as GSDMD‐CT did in the native protein. Upon light stimulation, the conformational changes in cpLOV2, like those in WT LOV2, might lead to the uncaging of GSDMD‐NT to restore its function (Figure 4a). In the dark, the prototypic construct GSDMD‐NT‐cpLOV2 exhibited high cytotoxicity (Figure S5c,d, Supporting Information), indicating the high potency of GSDMD‐NT and the necessity to mitigate its basal activity at rest. To overcome this hurdle, we created several mutants by neutralizing positive charges in the putative pore‐forming region of GSDMD‐NT with alanines (Figure 4b; bottom). Among the constructs tested using the previously‐mentioned SYTOX Blue and Annexin V staining as readouts, we identified a 4A mutant (R138A/K146A/R152A/R154A; designated LiPOP2b) that showed low cytotoxicity in the dark but regained potent cytotoxicity in the lit state (38.1%; Figure 4c,d, bottom; Figure S5c,d, Supporting Information). Again, the light‐inducible pyroptosis mediated by LiPOP2b was validated independently by time‐lapse confocal imaging (Figure 4f; Movie S7, Supporting Information) and LDH release (Figure 4h).

To further gain a mechanistic view on the action of LiPOP2b, we performed a lipid–strip assay by incubating recombinant LiPOP2b (Figure S7a, Supporting Information) with varying immobilized phospholipids. We found that the recombinant LiPOP2a showed light‐dependent interaction with PM‐enriched lipids, such as PI(3,5)P_2_, PI(4,5)P_2_, and PI(3,4,5)P_3_, in response to blue light stimulation (Figure 4i). Interestingly, upon blue light stimulation, LiPOP2b also interacted with PI3P (enriched in endosomes) and PI4P (enriched in Golgi and PM),^[^ ^29^ ^]^ implying that GSDMD‐NT may have membrane‐perforating activities toward subcellular organelles; an interesting direction warrants future investigation. Taken together, capitalizing on light‐inducible protein–protein dissociation and photo‐inducible uncaging, we have recapitulated key signaling steps during pyroptosis.

### 2.6 Optogenetic Control of Bacteria Killing and Leukemia Cell Suicide

**Figure 5:**
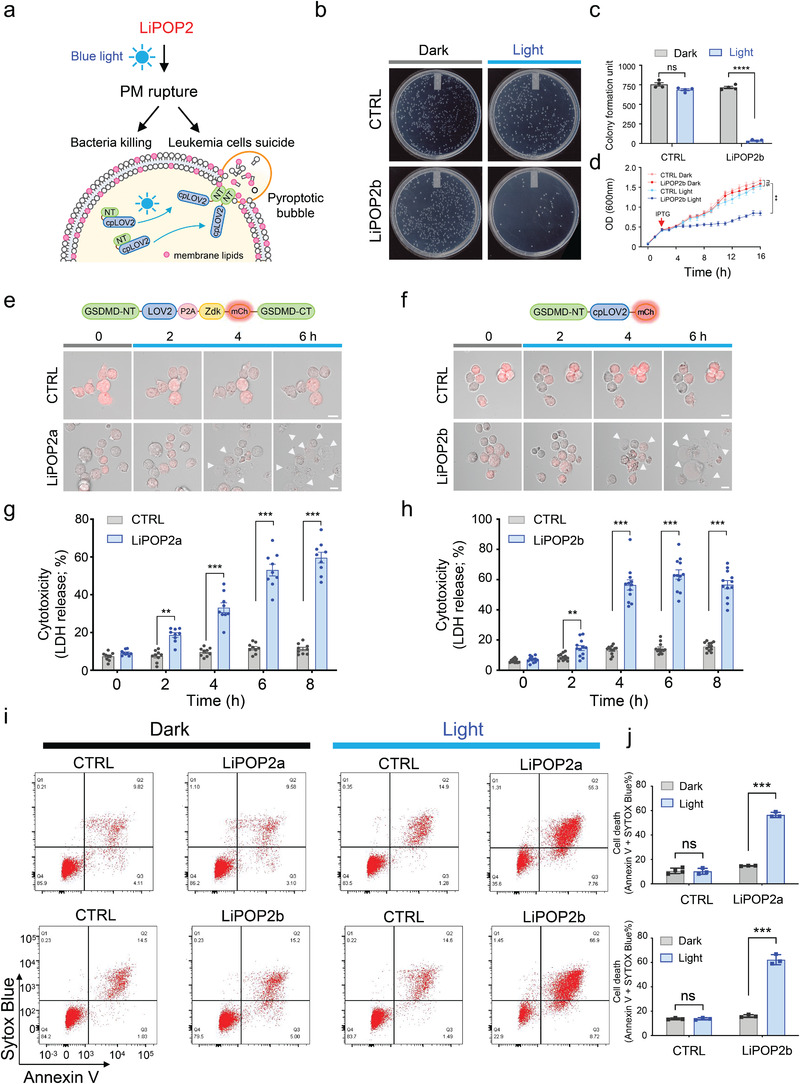
Optical control of bacteria killing and leukemia cell suicide. Photostimulation was applied at 470 nm at a power density of 4 mW cm^−2^ or using a 488‐nm confocal laser (5% output). ** *P* < 0.01; ***P < 0.001 when compared to the corresponding dark group (two‐tailed Student's t‐test). ns, not significant. Scale bar, 10 µm. a) Cartoon illustration of light‐inducible killing of bacteria and leukemia cells with LiPOP2. b–d) LiPOP2b exerts light‐dependent cytotoxicity to bacteria. The plasmid encoding LiPOP2b was transformed into the BL21 *E.coli* cells. Cells were spread on LB plates containing 0.5  x 10^–3^ m IPTG to induce LiPOP2b expression. The plates were either shielded or exposed to pulsed blue light for 16 h (10 s ON + 50 s OFF) at 37 °C (b). Quantification of colony‐forming units before or after blue light stimulation (c, upper panel). OD_600_ value was measured at the indicated time points for bacterial growth (d). *n* = 3 (mean ± s.d.). e,f) Time‐lapse confocal imaging of Jurkat CAR T‐cells stably expressing LiPOP2a (e) or LiPOP2b (f) upon blue light illumination for 6 h. White arrowheads indicate pyroptotic bubble formation. Also see Movies S8 and S9, Supporting Information. g,h) Quantification of LDH release cells expressing LiPOP2a (g) or LiPOP2b (h) at the indicated time‐points following photostimulation. Data were presented as mean ± s.d. from nine independent assays. i) Cell viability analysis on Jurkat CAR T‐cells stably expressing LiPOP2a, LiPOP2b, and their corresponding controls (LOV2‐P2A‐Zdk‐mCh‐GSDMD‐CT for LiPOP2a; cpLOV2‐mCh for LiPOP2b) by flow cytometry. Annexin V and SYTOX Blue staining were used to detect pyroptotic cells. The cells were either shielded or exposed to pulsed blue light for 6 h. j) Quantification of the percentage of double positive cells expressing LiPOP2a (top) or LiPOP2b (bottom). Data were presented as mean ± s.d. from three independent assays.

We further moved on to test the application of LiPOP2 in two biological contexts: the killing of prokaryotic cells and leukemia cell suicide (**Figure** **5**a). Because GSDMD‐NT has been shown to bind cardiolipin, a major lipid found in the bacterial membrane, to kill bacteria,^[^ ^30^ ^]^ we reasoned that inducible expression of LiPOP2b will likewise have antibacterial activity in the host bacteria strain when exposed to light stimulation. To test this, we subcloned LiPOP2b into an IPTG‐inducible pTriEx vector, and transformed the plasmid into BL21(DE3) *Escherichia coli* cells, which were either shielded from light or exposed to blue light illumination during overnight growth (Figure S7b,c, Supporting Information). The bacterial colony‐forming units were significantly reduced in the presence of IPTG to induce LiPOP2b expression (Figure 5b,c; and Figure S7b,c, Supporting Information). In addition, the growth of bacteria transfected with LiPOP2b in liquid LB medium was blocked in response to blue light stimulation after IPTG induction (Figure 5d). By contrast, the control group showed no light‐dependent effects (Figure 5b–d). Clearly, LiPOP2b can be used as a genetically‐encoded bactericide to eliminate bacteria in a light‐dependent manner.

Immunomodulation has become a central element in cancer therapies and graft‐versus‐host disease caused by transplantation of allogeneic organs. However, pathological immune responses and toxicities associated with vector/transgene integration and infused or donor cells themselves limit their clinical applications. Standard immunosuppressive drugs do not specifically target engineered or alloreactive T cells. Suicide genes have been exploited to selectively deplete transgenic T cells. Two suicide genes, inducible caspase‐9 (iCasp9)^[^ ^31^ ^]^ and herpes simplex virus type 1 thymidine kinase (HSV‐TK),^[^ ^32^ ^]^ are currently under clinical trials, with their utilities being demonstrated in haploidentical hematopoietic stem cell transplantation.^[^ ^33^ ^]^ However, a practical limitation of iCasp9 and HSV‐TK is the requirement for an additional drug that may not efficiently infiltrate the solid tumor microenvironment,^[^ ^31^, ^34^ ^]^ or under some conditions, may impose undesired side effects on endogenous cell signaling to cause nonspecific immune reactions. There remains a clinical need to develop safe and efficient methods to remove adoptively transferred T cells with high spatiotemporal precision. To explore alternatives, we introduced the light‐inducible suicide gene LiPOP2 into Jurkat T cells engineered to express chimeric antigen receptors (CARs). In either LiPOP2a‐ or LiPOP2b‐expressing Jurkat CAR T‐cells, we observed an efficient induction of cell death with pyroptotic morphology, accompanied with LDH release to reflect the time‐dependent increase of cellular component release in response to blue light stimulation (*t*_1/2_: 4.0 ± 0.3 h for LiPOP2a and 3.0 ± 0.3 h for LiPOP2b; Figure 5e–h; Movies S8 and S9, Supporting Information). These findings were quantitatively confirmed by flow cytometry analysis of cell death using SYTOX Blue and Annexin V staining (Figure 5i,j). The lit group showed an ≈four to fivefold increase of cell death upon blue light stimulation within 6 h. By contrast, the control showed no significant light‐inducible effects in all three assays (Figure 5e–j). In aggregate, our findings set the stage for future exploration of LiPOP as an alternative solution for selective eradiation of engineered T cells using light.

### 2.7 Discussion and Conclusion

In this study, we have faithfully reconstructed the key molecular steps involved in non‐apoptotic cell death pathways (necroptotic and pyroptotic signaling) by taking an optogenetic engineering approach. During this process, we have designed and optimized a set of optogenetic tools, designated LiPOPs, to remotely control necroptosis and pyroptosis without co‐activation of apoptotic signaling that may arise from pathophysiological stimuli. Compared to chemically‐inducible apoptosis tools derived from caspase 9 and HSV‐TK,^[^ ^34b^ ^]^ LiPOP excels in its superior temporal and spatial resolution, and rapid activation kinetics (Table S2, Supporting Information). Reactive oxygen species (ROS), generated by fluorescent proteins or photosensory domains upon irradiation, have also been found to be effective for initiating cell‐ablation in bacteria,^[^ ^35^ ^]^ eukaryotic cells,^[^ ^35^, ^36^ ^]^ zebrafish,^[^ ^37^ ^]^ and *Caenorhabditis elegans*.^[^ ^38^ ^]^ Representative approaches using light‐induced production of ROS by chromogenic tools or chemical compounds include KillerRed,^[^ ^35^, ^36^, ^37^ ^]^ SuperNovaGreen,^[^ ^36b^ ^]^ and miniSOG.^[^ ^38^ ^]^ These tools take effect via an apoptotic pathway by targeting mitochondria or through membrane lipid oxidation using a membrane‐localized photosensitizer (Table S3, Supporting Information). Given that ROS can be freely diffused into neighboring cells to mediate a wide range of biological processes, these photosensitizers tend to cause undesired off‐target effects. A side‐by‐side comparison among the key parameters of major cell‐ablation or suicidal devices is presented in Table S3, Supporting Information.

A multitude of potential applications for LiPOPs can be predicted in different fields of biology and medicine. LiPOPs are capable of killing mammalian cells in culture and in mouse models. Potentially, this approach could be expanded to allow light‐driven spatially‐ and temporally‐controlled ablation of specific cell types in the developing embryo. Many important model animals are either sufficiently small or translucent to allow light penetration, and therefore, are suitable for optogenetic manipulations. To make these tools amenable for manipulating deeply buried tissues, we have illustrated herein two wireless optogenetic approaches: (i) coupling with nanomaterials (UCNPs) to enable NIR‐to‐blue emission, thereby permitting indirect photo‐activation of optogenetic constructs with a relatively deeper tissue penetration; (ii) the use of a NanoLOGS platform that utilizes bioluminescence as a light source to effectively activate LiPOPs for tumor lysis in living animals (Table S4, Supporting Information). The latter strategy will probably sacrifice the spatial resolution of optogenetic tools, but has the benefit of extending optogenetic applications to nearly any tissues or organs that can be reached by the substrate.

During necroptosis and pyroptosis, the formation of pores causes cell membrane rupture and release of cytokines and various damage‐associated molecular pattern molecules, which eventually results in an inflammatory cascade. Therefore, LiPOP‐mediated cancer cell death has the potential to improve the efficacy of cancer immunotherapies by increasing lymphocyte infiltration and/or activation within the tumor microenvironment (TME).^[^ ^11a,b,c^ ^]^ The LiPOP tools generated in this study will likely find future applications in the mechanistic dissection of how necroptosis and pyroptosis remodel the TME to benefit cancer therapies. From a translational perspective, LiPOPs can also be repurposed as a synthetic light‐switchable gene that allows selective elimination of therapeutic cells after adoptive transfer (such as CAR T‐cell therapy^[^ ^39^ ^]^), as well as used as a genetically‐encoded bactericide to induce bacteria killing with a pulse of light.

## 3 Experimental Section

### Reagents and Antibodies

Furimazine (Fz) as the NanoLuc luciferase assay substrate was purchased from Promega (#N1110). FFz was originally developed by the Michael Z. Lin group at Stanford University and obtained from Promega. UCNPs were generated as described previously.^[^ ^19^ ^]^CytoTox 96 non‐radioactive cytotoxicity assay kit was bought from Promega (#G1781). Enhanced chemiluminescence (ECL) Western blotting substrate was purchased from Thermo Fisher Scientific (#32106). Isopropyl *β*‐D‐1‐thiogalactopyranoside (IPTG, #367‐93‐1) and KOD Hot Start DNA polymerase (#71086‐4) were purchased from Sigma. The T4 DNA ligase kit (#M0202M) and NEBuilder HiFi DNA Assembly Master Mix (M5520AA) were purchased from New England BioLabs. QuikChange Multi Site‐Directed Mutagenesis Kit (#210513) was obtained from Agilent Technologies. Pacific Blue Annexin V (NC9818309) was from Biolegend. FACS staining antibodies, FITC Annexin V (51‐65874X), and SYTOX Blue (S11348), were from BD Biosciences and Invitrogen, respectively. Mouse monoclonal anti‐FLAG (F3165) antibody was purchased from Sigma. The rabbit polyclonal anti‐mCherry (NBP2‐25157) antibody was obtained from Novus Biologicals. The rabbit polyclonal anti‐full length GFP (sc‐8334) and anti‐GAPDH (sc‐25778) antibodies were obtained from Santa Cruz Biotechnology. Secondary antibodies, including goat anti‐mouse IgG–HRP (sc‐2005) and goat anti‐rabbit IgG‐HRP (sc‐2004), were purchased from Santa Cruz Biotechnology. pRIPK3‐S227 (93654S), pMLKL‐S358 (91689S), and MBP‐Tag (2396S) antibodies were from Cell Signaling Technology.

### Plasmids

pGP‐CMV‐GCaMP6s‐CAAX (#52228), pUAS‐NanoLuc (#87696), packing vectors pMD2.G (#12259), and psPAX2 (#12260), and the lentiviral vector pWPXL (#12257) were obtained from Addgene. mCherry‐CRY2‐RIPK1 and mCherry‐CRY2‐RIPK3 were generated by inserting the cDNAs encoding RIPK1 (#78842, Addgene), RIPK3 (#78822, Addgene) into a home‐made vector mCherry‐CRY2_PHR_ modified from pmCherry‐C1. To generate the constructs for MLKL‐mediated necroptosis, MLKL N‐terminal truncations were generated by inserting the cDNA encoding human MLKL (#106078, Addgene) into the mCherry‐CRY2_PHR_ vector using the NheI site. MLKL (1‐125)‐mCherry‐CRY2 mutations were generated by using the QuikChange Lightning Multi site‐directed mutagenesis kit (Agilent). LiPOP1‐NanoLuc was made by inserting Nanoluc (#87696, Addgene) into LiPOP1 between mCherry and CRY2_PHR_ by using the AgeI restriction site. To make a stable cell line expressing LiPOP1‐NanoLuc, LiPOP1‐NanoLuc and the corresponding control vector, mCherry‐NanoLuc‐CRY2_PHR_, were amplified via standard PCR and then inserted into a modified pLentiviral vector (#61425, Addgene). The insert was digested by BspEI and BamHI, and the vector by AgeI and BamHI. To generate the constructs for GSDMD‐mediated pyroptosis, mouse GSDMD (1‐276, #80950, Addgene) was amplified by standard PCR. The circularly permuted LOV2 (cpLOV2) was synthesized as gBlock with T517 as the new terminus and inserted into mCherry2‐N1 between restriction sites HindIII and EcoRI to make cpLOV2‐mCh. For LiPOP2b, mouse GSDMD (1‐276) was inserted into a cpLOV2‐mCherry plasmid between the NheI and HindIII sites. GSDMSD (1‐276)‐related mutations were made by using the QuikChange Lightning Multi site‐directed mutagenesis kit (Agilent). For LiPOP2a, cDNAs encoding the “LOV2‐P2A‐Zdk‐mCherry” element were synthesized as gBlock and inserted into FLAG‐GSDMD (#80950, Addgene) between residues 276 and 277 by using the NEBuilder HiFi DNA Assembly Master Mix. To generate stable cell lines, mGSDMD1‐276(4A)‐cpLOV2‐mCherry and its control cpLOV2‐mCherry, and FLAG‐mGSDMD‐NT‐LOV2‐P2A‐Zdk‐mCherry‐mGSDMD‐CT and its control LOV2‐P2A‐ Zdk‐mCherry‐mGSDMD‐C, were inserted into pWPXL (#12257, Addgene) between the PmeI and SpeI sites. To generate bacterial expression constructs, mGSDMD1‐276(4A)‐cpLOV2‐mCherry and its control cpLOV2‐mCherry were cloned into a modified pTriEX vector (#73614, Addgene) using BglII and XhoI restriction sites. LiPOP tools are available from Addgene (LiPOP1, #168264; LiPOP1‐NanoLuc, #168265; LiPOP2, #168266). For protein purification, pMAL‐c5X (#66998, Addgene) was modified as pMAL‐c5X‐MBP‐MCS by using the NEBuilder HiFi DNA Assembly Master Mix. Then, LiPOP2b or its control cpLOV2‐mCherry was inserted using the NotI and HindIII sites. The CAR components (CD8 signal peptide, Myc tag, anti‐CD19 scFv, CD8‐alpha transmembrane domain, hinge region, 4‐1BB, and CD3z) were synthesized as gBlock by Integrated DNA Technologies (Coralville, Lowa, USA) and inserted into a modified pLentiviral vector (#61425, Addgene) using HpaI and PacI restriction sites. Plasmid DNA was purified using Omega Bio‐tek mini kit (DNACOL‐02) for use in cell‐based assays. All constructs were verified via diagnostic digestion and Sanger DNA sequencing.

### Cell Transfection

Transient transfection of HeLa and HEK293T cells was performed using Lipofectamine 3 000 (Invitrogen) reagent by following the manufacturer's instructions. For stable expression, lentiviral plasmids (pWPXL or pLentiBlast) harboring the desired gene were first transfected by iMFectin (#I7200, GenDePOT) into HEK293T cells together with the packing plasmids psPAX2 and pMD2G with a ratio of 5:3:2 for 48 h. Then, the supernatants were collected and used to infect B16, HeLa, or 786‐O cells for another 48 h. For adherent cell lines, mCherry‐positive cells were manually picked as single clones under fluorescence microscope. mCherry‐positive transduced engineered T cells were sorted by flow cytometry using a FACSFusion cell sorter (BD Biosciences). Single clones were further expanded for 2–4 additional weeks to establish stable cell lines.

### Immunoblotting

The cells were washed in chilled PBS three times and lysed directly using a CST lysis buffer (20 x 10^–3^ m Tris‐HCl (pH 7.5), 150 x 10^–3^ m NaCl, 1 x 10^–3^ m EDTA, 1 x 10^–3^ m EGTA, 1% Triton X‐100, 2.5  x 10^–3^ m Na pyrophosphate, and 1 x 10^–3^ m *β*‐glycerophosphate, pH7.5) for 30 min at 4 °C. The lysis buffer contained 1X protease inhibitor cocktail (P3100‐010, GenDEPOT) and phosphatase inhibitor cocktail (P3200‐001, GenDEPOT). After centrifugation at 20 000 g at 4 °C for 10 min, the supernatant was collected, with the total protein concentrations determined by a BCA protein assay (#23225, Thermo Scientific). For immunoprecipitation, the cell lysates were incubated with the indicated antibodies and magnetic A/G beads (#88803, Thermo Scientific) overnight at 4 °C. The beads were then pelleted and washed with lysis buffer five times. 1X denaturing loading buffer was added and heated for 10 min at 95 °C before loading into SDS‐PAGE gels. Cell lysates were electrophoretically separated on 8–16% SDS‐PAGE (M00660, GenScript), followed by transfer onto a nitrocellulose blotting membrane (A15735264, GE Healthcare, Life science) and probed with appropriate antibodies.

### Flow Cytometry

For flow cytometric quantification of cell death and cell viability, the cells were treated as described in the text. Cells were detached by trypsin (adherent cell lines) or simply collected (suspension cell lines), washed with cold PBS three times and stained using FITC Annexin V (#556420, BD Biosciences) and SYTOX Blue (S34857, Thermo Fisher) according to the manufacturer's instructions. Briefly, 1 × 10^5^ cells were resuspended in 100 µL 1X binding buffer and incubated with 5µL FITC Annexin V and SYTOX Blue (1 µm final concentration) for 15 min at RT in the dark. Stained cells were analyzed using a LSRII flow cytometer (BD Biosciences) and data were processed using the FlowJo software.

### Confocal Imaging

To examine cytotoxicity of constructs and the morphology of necroptotic and pyroptotic cells, HeLa cells were plated on glass‐bottomed dishes (#D35‐20‐0‐TOP, Cellvis). The cells were then transfected with indicated plasmids using Lipofectamine 3 000 (Invitrogen). After transfection for 24h, samples were observed and captured on a Nikon Ti2 Inverted microscope with a Yokogawa W‐1 dual spinning disk scanhead, Micro‐Scanner for photo‐stimulation and stage top incubator for live‐cell imaging. The system is equipped with six solid‐state lasers (405 nm, 445 nm, 488 nm, 514 nm, and 640 nm) for imaging and one additional laser for optogenetics (473 nm). Multichannel images were captured sequentially using 405, 488, and 561nm excitation wavelengths and the appropriate single emission filters (460, 525, and 600 nm, respectively) and captured using a Photometrics Prime BSI, back‐side illuminated sCMOS camera. For time‐lapse imaging, the indicated stable cells were seeded on glass‐bottomed dishes at about 30–40% confluency for 16 h. A 60 × oil lens was used for high‐resolution images and image capture was conducted with an Oko labs Stage top incubator, which provides incubation with full environmental control including heating, humidity and CO_2_ regulation. For Jurkat CAR T‐cells, the plates were coated with poly‐L‐lysine (#2840311, Millipore) before seeding the cells. Blue light irradiation was performed by the LED light source (470 nm, 4 mW/cm^2^, ThorLabs Inc.) or the built‐in 488‐nm laser source (5% input). To monitor Ca^2+^ influx in response to blue light stimulation, pGP‐CMV‐GCaMP6s‐CAAX (#52228, Addgene) was transfected in HeLa cells stably expressing LiPOP1. For measurement of Ca^2+^ influx, 488 nm and 561 nm laser sources were used to excite GFP in pGP‐CMV‐GCaMP6s‐CAAX and mCherry in LiPOP1, respectively, at an interval of 30 seconds. The captured images were analyzed by the NOS‐Elements AR microscope imaging software (Nikon, NIS‐element AR version 4.0) or ImageJ program. Dozens of cells were selected to define regions of interest (ROI) for analysis. All image data shown are representative of at least three times.

### Cytotoxicity Assays

Cell death and cell viability were determined by the LDH release assay using the CytoTox 96 non‐radioactive cytotoxicity assay kit (#G1781, Promega) according to the manufacturer's protocol. Briefly, cells (4000 cells per well for HeLa and 8000 cells per well for Jurkat CAR T‐cells) were seeded in 96‐well plates and cultured for 24 h at 37 °C before treatment. For Maximum LDH Released, cells were lysed by adding 10 µL of lysis solution per 100 µL of medium and incubated for 30 min. For experimental LDH release, upon blue light stimulation for indicated time points, 50 µL of supernatant from all test and control wells were collected and transferred to a fresh 96‐well flat‐bottom plate. Subsequently, 50 µL of the CytoTox 96 reagent were added and incubated for 30 min in the dark at room temperature. Next, 50 µL stop solution was added into each well for termination of the reaction. The absorbance at 490 nm was recorded by using BioTek Synergy Neo2. 50 µL culture medium was set and tested as the baseline LDH activity. The formula for computing percent cytotoxicity: Percent cytotoxicity = 100 x (Experimental LDH release–Baseline LDH [OD_490_]/maximum LDH release–Baseline LDH [OD_490_]).

### Purification of Recombinant LiPOP2b Proteins

DNA encoding cpLOV‐mGSDMD (1‐276) was amplified by standard PCR, and subcloned into pMAL‐c5X‐MBP‐MCS (mentioned in Plasmids). The plasmid was transformed into *E. coli* strain BL21 (DE3) cells. The cells were grown in LB medium supplemented with 100 mg mL^−1^ ampicillin (#171358, Fisher Scientific). Protein expression was induced overnight at 16 °C with 0.6 x 10^–3^ m IPTG. When OD_600_ reached 0.6–0.8, cells were harvested after centrifugation. The cell pellets were resuspended in 1 × PBS (pH 7.4) and subjected to pulsed sonication. Cell lysates were clarified by centrifugation at 4 °C and supernatants were loaded onto Ni^2+^‐nitrilotriacetic acid (Ni‐NTA)‐agarose resin (Qiagen). After extensive washing with PBS buffer containing 25 x 10^–3^ m imidazole for three times, the bound protein was eluted with PBS containing 250 x 10^–3^ m imidazole and 1 x 10^–3^ m TCEP. Eluted fractions were further purified by gel filtration with a Superdex 200 10/300 GL column using the AKTApure fast protein liquid chromatography system (GE Healthcare).

### Protein–Lipid Binding Assay

Cell lysates (LiPOP1, 0.5 µg mL^−1^) or purified recombinant proteins (LiPOP2b, 0.5 µg mL^−1^) were incubated with the PIP Strip membrane (#P‐6001, Echelon Biosciences) and gently agitated at room temperature for 60 min with or without blue light illumination (470 nm, 4 mW cm^−2^). To block non‐specific binding, the membranes were preincubated with 3% fatty acid‐free BSA in PBS‐T (0.1% v/v Tween 20) for 1 h at room temperature. The membrane‐bound proteins were detected by a Rabbit mCherry (for LiPOP1) or mouse anti‐MBP (for mGSDMD (1‐276) 4A‐cpLOV2, #2396s, CST) antibody diluted at 1:1 000 for 1 h at room temperature. After extensive washing, the membrane was incubated with horseradish peroxidase‐conjugated anti‐rabbit antibody (1:2 000) for 1 h at room temperature with gentle agitation, followed by washing in PBS‐T. Proteins were detected by using ECL Western Blotting Substrate (32106, Thermo Scientific).

### Bacterial Colony Formation Assay

To assay the cytotoxicity of LiPOP2b in prokaryotes, BL21 (DE3) *E. coli* cells were transformed with 0.5 x 10^–3^ m of LiPOP2b DNA. The transformed cells were serially diluted and plated onto LB agar containing the appropriate antibiotics in the presence or absence of IPTG. The colony‐forming unit was determined by counting the number of viable bacteria per transformation after 16 h at 37 °C with or without blue light irradiation. For growth curve of bacteria in liquid LB medium, when the OD_600_ value reached 0.4, IPTG was added to a final concentration of 0.5 x 10^–3^ m. OD_600_ values were measured every hour for 14 h with or without blue light illumination.

### Mouse Xenograft Models and *In Vivo* Bioluminescence Imaging

All animal studies were approved by the Institutional Animal Care and Use Committee (IACUC) at the Institute of Biosciences and Technology, Texas A&M University or University of Massachusetts Medical School. On day 0, 8 week‐old female immunodeficient mice (scid‐beige mice, CBSCBG‐F from Taconic Biosciences) were inoculated intradermally with 1) 10^6^ HeLa cells stably expressing LiPOP1 or mCh‐CRY2 (as control) for UCNP‐mediated tumor cell killing *in vivo*; or 2) 10^6^ 786‐O cells stably expressing LiPOP1‐NanoLuc or mCh‐NanoLuc‐CRY2 (as control) for bioluminescence‐ mediated tumor cell killing *in vivo*. For the UCNP‐mediated tumor cell killing group, 150 µg of UCNPs were injected into each tumor site of SCID‐beige mice 9 days after inoculation. Mice were subjected to pulsed NIR light stimulation (980 nm at a power density of 250 mW cm^−2^; 20 s ON + 5 min OFF; 2h every three days). To avoid potential skin burns, Vaseline was applied before NIR light stimulation. A 980 nm laser resource was used for photostimulation (Model MDL‐980; Changchun New Industries Optoelectronics Technology). For the bioluminescence‐mediated tumor cell killing group, 1.3 µmol FFz was i.p. injected into mice every 3 days (a total of seven doses). Bioluminescence of tumors was measured by an IVIS Spectrum *in vivo* imaging system at day 9 and day 30 after tumor inoculation. Tumor size was measured at indicated time points by using digital calipers with the formula: tumor volume = length × width^2^/2 (mm^3^). At day 30, mice were euthanized for tumor isolation and phenotypic analyses.

### Evaluation of *In Vivo* Toxicity of FFz

8‐week‐old Scid‐beige mice were i.p. injected with FFz (for 25‐g mouse 1.3 µmol every 3 days; a total of seven doses). At day 21, mice were sacrificed and histological and routine blood analysis was performed. The mice i.p. injected with PBS were used as control. Major organs (heart, liver, spleen, lung, kidney, and muscle) were dissected for H&E staining. Before the mice were euthanatized, blood samples (≈0.5 mL) were collected for a routine complete blood count (CBC) test by using the VETSCAN HM5 Hematology Analyzer (Abaxis, Inc., CA, USA).

### UV–Vis Spectra Measurements

The Nanodrop 2000 spectrophotometer (Thermo Scientific, Waltham, MA, USA) was used to record UV–vis absorbance spectra for cpLOV2. Purified cpLOV2 protein (in buffer 20 x 10^–3^ m Na_2_HPO_4_/NaH_2_PO_4_, 50 x 10^–3^ m NaCl at pH 6.2) was concentrated to 3 mg mL^−1^ and subjected to 470 nm blue light excitation (4 mW cm^−2^, 2 min). The absorbance spectra at wavelengths between 410 and 540 nm was acquired immediately after blue light stimulation and then the signals were recorded every 25 s until the cpLOV2 protein fully recovered from the lit state (blue) to the dark state (black). This result indicated that circular permutation did not disrupt the photochemical property of cpLOV2.

### Statistical Analysis

Quantitative data are expressed as mean ± standard error of the mean (s.e.m.) or mean ± standard error (s.d.). Statistical significance was determined with two‐tailed paired Student's *t*‐test. ns, not significant; ∗*P* < 0.05; ∗∗*P* < 0.01; ∗∗∗*P* < 0.001; ∗∗∗∗*P* < 0.0001, when compared with control.

## Conflict of Interest

The authors declare no conflict of interest.

## Author Contributions

Y.Z. and J.J. conceived the ideas and directed the work. Y.Z., G.H., and J.J. designed the study. L.H., Z.H., K.H., R.C., N.T.N., R.W., X.C., and Z.H. performed the experiments. J.J. and X.C. analyzed the results. Y.Z. and J.J. wrote the manuscript. S.S. edited the manuscript. J.W. synthesized the flurofurimazine (FFz). All of the authors contributed to the discussion and editing of the manuscript.

## Supporting information

Supporting Information

Supplemental Movie 1

Supplemental Movie 2

Supplemental Movie 3

Supplemental Movie 4

Supplemental Movie 5

Supplemental Movie 6

Supplemental Movie 7

Supplemental Movie 8

Supplemental Movie 9

## Data Availability

The data that support the findings of this study are available from the corresponding author upon reasonable request.
