# Supplementary material for: Optogenetic Control of Non‐Apoptotic Cell Death
Source: Adv Sci (Weinh). 2021 May 6;8(13):2100424. doi: 10.1002/advs.202100424 (PMC8438606; doi:10.1002/advs.202100424)
Supplement: Supplementary file 1 — Supporting Information [file ADVS-8-2100424-s003.pdf]

© 2021 Wiley-VCH GmbH

ADVANCED SCIENCE
Open Access

Supporting Information

for *Adv. Sci.,* DOI: 10.1002/advs.202100424

Optogenetic control of non-apoptotic cell death

Lian He, Zixian Huang, Kai Huang, Rui Chen, Nhung T. Nguyen, Rui Wang, Xiaoli Cai, Zhiquan Huang, Stefan Siwko, Joel R. Walker, Gang Han, Yubin Zhou*, and Ji Jing**

Supplementary Materials for

Optogenetic control of non-apoptotic cell death

Lian He, Zixian Huang, Kai Huang, Rui Chen, Nhung T. Nguyen, Rui Wang, Xiaoli Cai, Zhiquan Huang, Stefan Siwko, Joel R. Walker, Gang Han, Yubin Zhou*, and Ji Jing**

This PDF file includes:

Supplementary Figures 1-7

Supplementary Tables 1-4

Captions for Supplementary Movies

Other Supplementary Materials for this manuscript includes the following:

Supplementary Movies 1-9

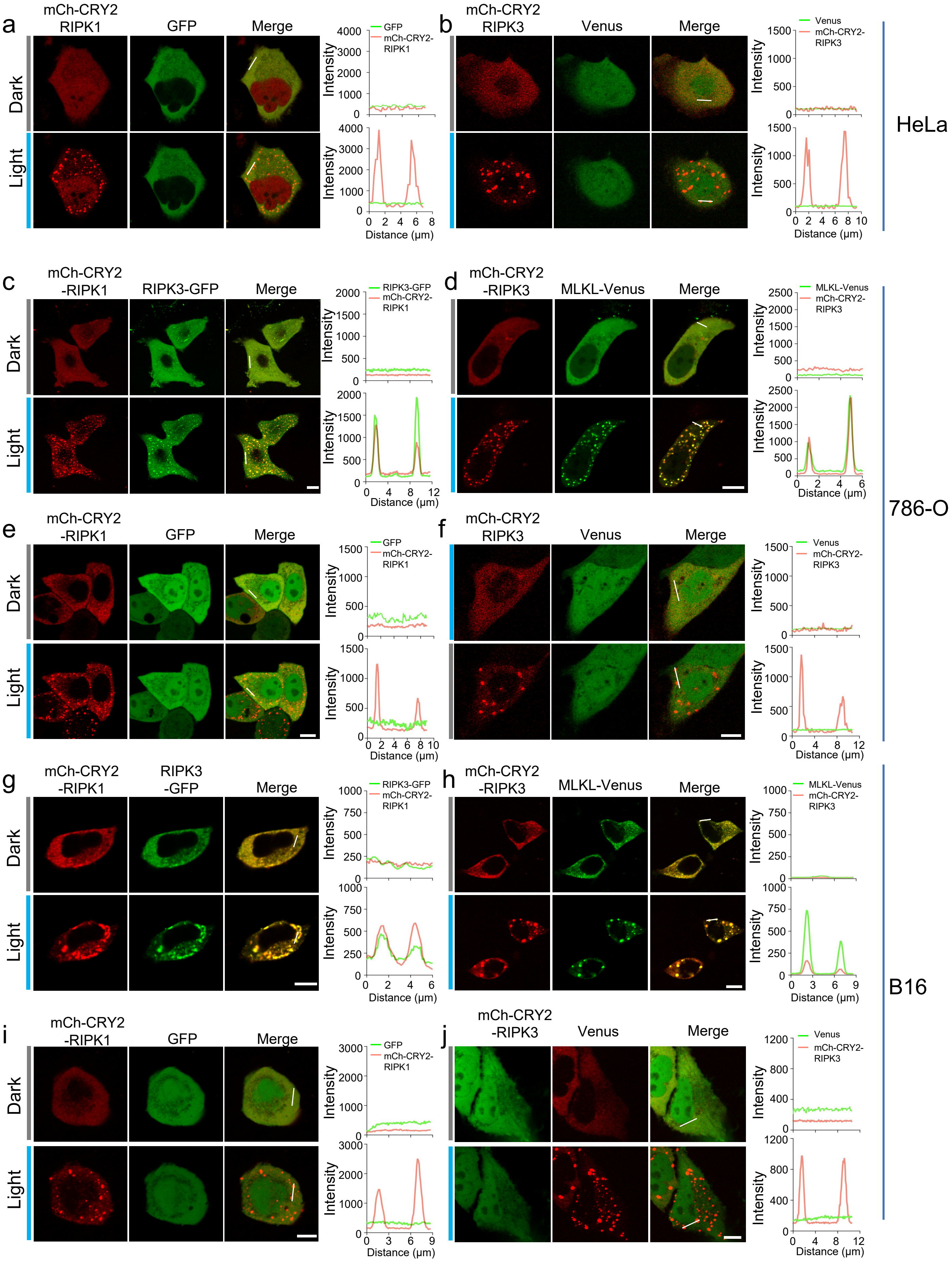

a
mCh-CRY2 RIPK1
GFP
Merge
Dark
Light
Intensity
Distance (μm)
b
mCh-CRY2 RIPK3
Venus
Merge
HeLa
c
mCh-CRY2 -RIPK1
RIPK3-GFP
d
mCh-CRY2 -RIPK3
MLKL-Venus
786-O
e
f
g
RIPK3 -GFP
h
B16
i
j

Figure S1 | Images of engineered optogenetic constructs in multiple cancer cell lines. (Related to **Fig. 1**) **a-b,** Representative confocal images of HeLa cells co-expressing (**a**) mCh-CRY2-RIPK1 (red) and GFP (green), or (**b**) mCh-CRY2-RIPK3 (red) and Venus (green) as negative controls, before and after blue light stimulation. The intensity profiles of GFP or Venus and mCh-CRY2-RIPK1 or mCh-CRY2-RIPK3 (across the white line) in response to blue light were plotted on the right. Also see **Supplementary Movies 1 and 2**. **c-j,** Representative confocal images of 786-O cells (**c-f**) or B16 cells (**g-j**) co-expressing (**c** or **g**) mCh-CRY2-RIPK1 (red) and RIPK3-GFP (green), or (**d** or **h**) mCh-CRY2-RIPK3 (red) and MLKL-Venus (green); (**e** or **i**) mCh-CRY2-RIPK1 (red) and GFP (green), or (**f** or **j**) mCh-CRY2-RIPK3 (red) and Venus (green) as negative control before and after blue light stimulation. The intensity profiles of GFP or Venus and mCh-CRY2-RIPK1 or mCh-CRY2-RIPK3 (across the white line) in response to blue light were plotted on the right (right panel). Scale bar, 10 μm.

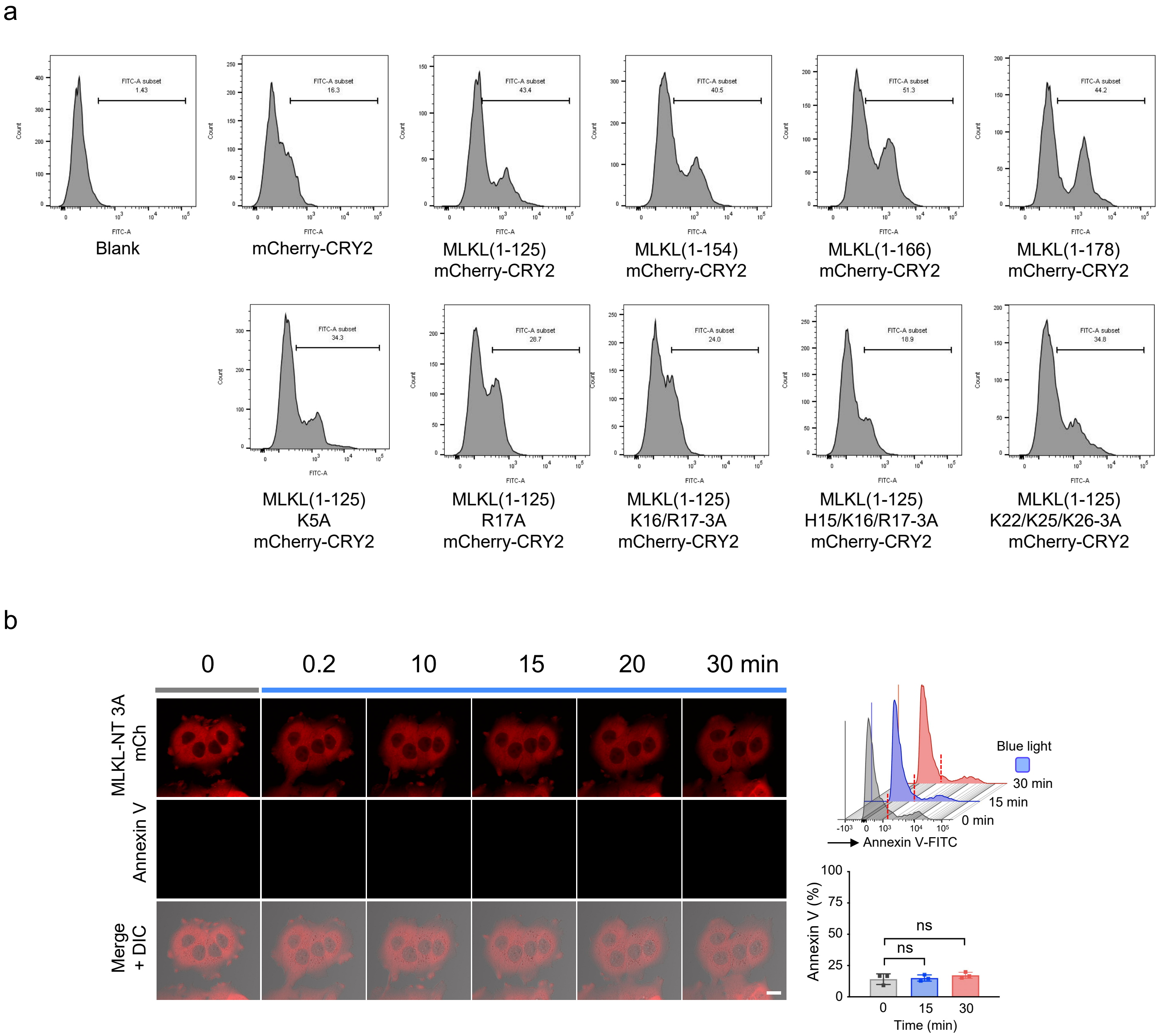

Figure S2 | Optimization of LiPOP1 constructs. (Related to **Fig. 1**)

a, Flow cytometry analysis of the basal activity of all the constructs listed in **Fig. 1g** prior to light illumination. Annexin V-FITC staining was used to assess the degrees of PM disruption due to necroptosis at 24 h post-transfection.

b, Representative confocal images of HeLa cells expressing MLKL-NT(3A)-mCh upon exposure to blue light (**left**). Pacific Blue Annexin V (blue) was an indicator for PS translocation from the inner half leaflet of PM to the outer membrane during necroptotic cell death. Light-induced necroptotic cell death assessed by flow cytometry (**right**). Annexin V-FITC was used to stain dying cells. n=3 (mean ± s.d.), ns, not significant (two-tailed Student's *t*-test).

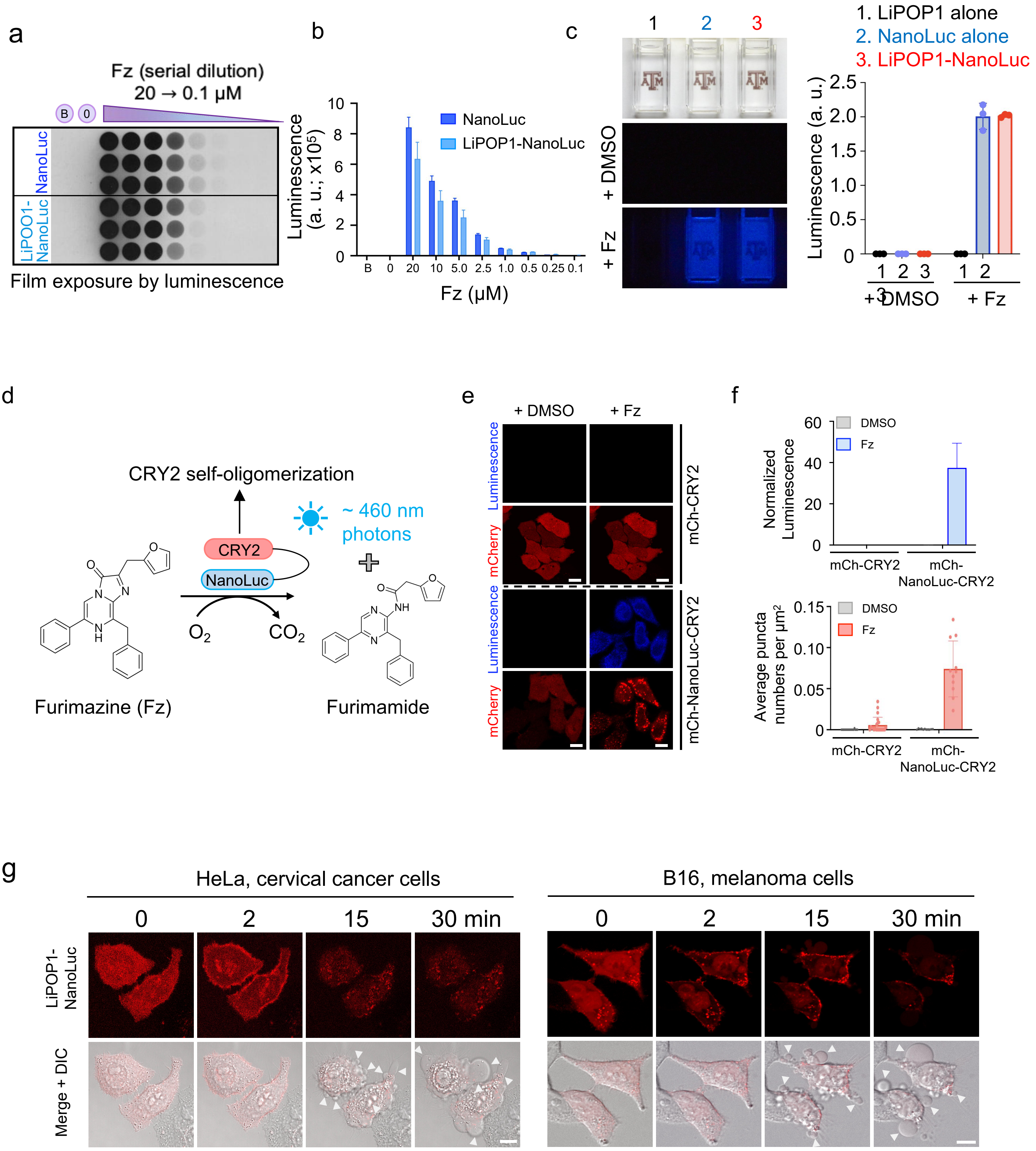
a
Fz (serial dilution)
20 → 0.1 μM
B
0
NanoLuc
LiPOO1-NanoLuc
Film exposure by luminescence
b
Luminescence (a. u.; x10^5)
NanoLuc
LiPOP1-NanoLuc
B 0 20 10 5.0 2.5 1.0 0.5 0.25 0.1
Fz (μM)
c
1 2 3
+ DMSO
+ Fz
1. LiPOP1 alone
2. NanoLuc alone
3. LiPOP1-NanoLuc
Luminescence (a. u.)
1 2 3 1 2 3
+ DMSO
+ Fz
d
CRY2 self-oligomerization
~ 460 nm photons
CRY2
NanoLuc
O2
CO2
Furimazine (Fz)
Furimamide
e
+ DMSO
+ Fz
Luminescence
mCherry
mCh-CRY2
mCh-NanoLuc-CRY2
f
Normalized Luminescence
DMSO
Fz
mCh-CRY2
mCh-NanoLuc-CRY2
Average puncta numbers per μm²
g
HeLa, cervical cancer cells
B16, melanoma cells
0 2 15 30 min
LiPOP1-NanoLuc
Merge + DIC

Figure S3 | NanoLOGS for optochemical activation of CRY2 and LiPOP1. (Related to **Fig. 3**)
a, A dot blot assay showing luminescence intensities of HeLa cells transfected with LiPOP1-NanoLuc or mCh-NanoLuc-CRY2 alone at 5 min after Fz treatment. An X-ray film was covered above a 96-well plate containing transfected HeLa cells to detect the luminescence in a dark room. B, blank.
b, Quantification of the results shown in panel **a**. n = 3 independent biological replicates.
c. Real-time visualization (left panel) and quantification (right panel) of bioluminescence in a clear cell adenocarcinoma cell line, 786-O. Tumor cells stably expressing LiPOP1-NanoLuc or NanoLuc alone were detached and resuspended in phenol-free cell culture media. The emission from the cell suspension was strong enough to lighten the background upon 10 μM Fz addition (bottom). DMSO used to dissolve Fz was used as control.
d. The working principle of NanoLOGS. NanoLuc catalyzes the conversion of Fz into furimamide with subsequent release of photons that emit at ~460 nm to initiate photoactivation of the CRY2 photoreceptors to cause self-oligomerization (puncta formation).
e-f, Representative confocal images of HeLa cells expressing mCh-CRY2 or mCh-NanoLuc-CRY2 in the absence (DMSO as control) or presence of Fz (10 μM). The normalized bioluminescent intensities (right top) and mCherry signals (right bottom) were shown next to the corresponding images. n=62 cells from three independent assays. Bioluminescence-aided photoactivation of of CRY2 could induce CRY2 homo-oligomerization to form puncta inside the cytosol. Data were shown as mean ± s.e.m.
g, Time-lapse confocal imaging of human HeLa cervical cancer cells (**left**) and mouse B16 melanoma cells (**right**) expressing LiPOP1-NanoLuc following the addition of 10 μM Fz at the indicated time points. White arrowheads indicated necroptotic bubble formation. DIC, differential interference contrast. Scale bar, 10 μm.

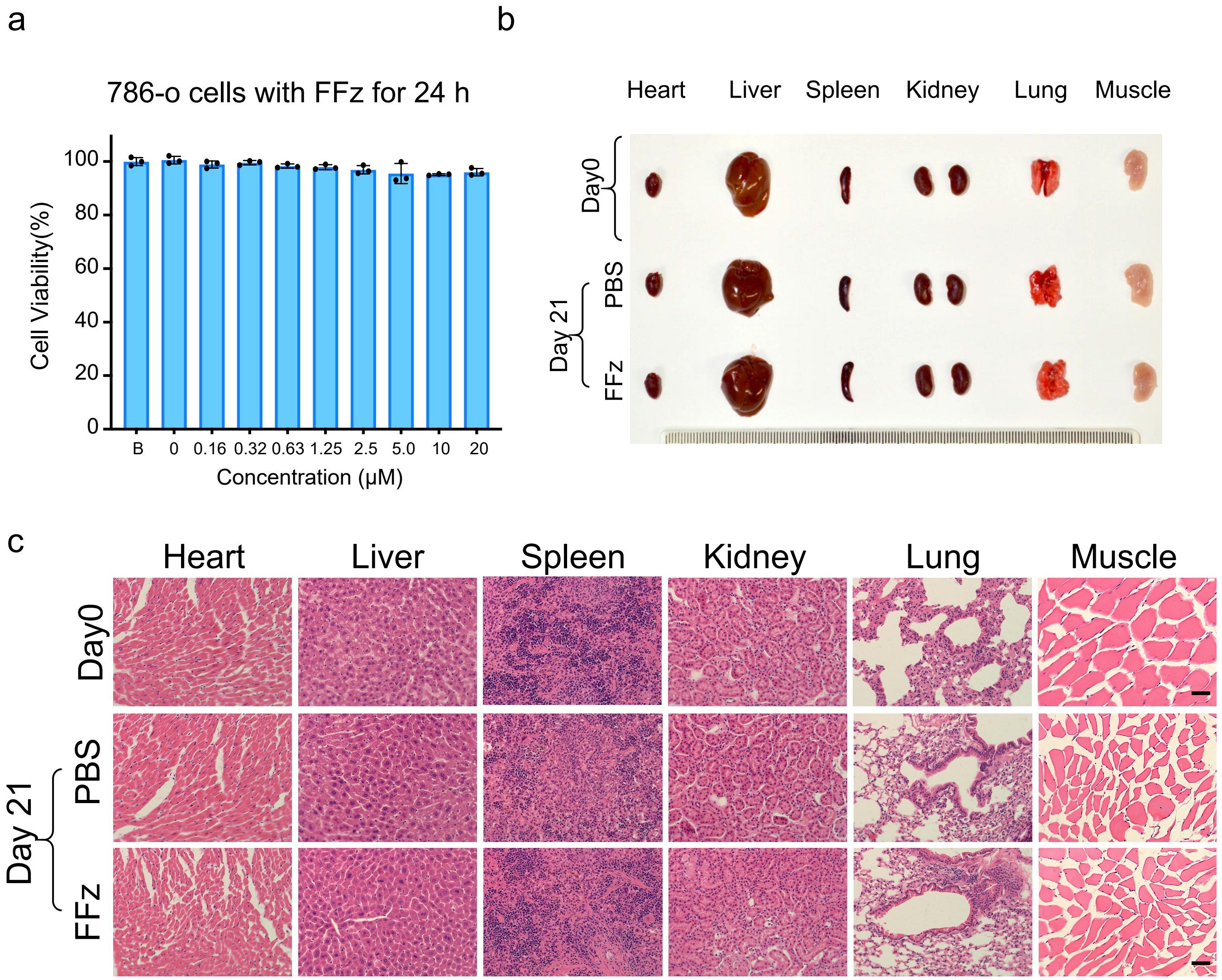

Figure S4 | *In vivo* biosafety evaluation of FFz. (Related to **Fig. 3**)

a, Assessment of 786-O cells viability at different concentrations of FFz for 24 hours.

b, Representative images of major organs isolated from the mice subcutaneously administered with PBS or 1.3 μmol (for 25-g mouse) FFz.

c. Typical Images of H&E staining of major organs shown in panel **b**. Scale bar, 100 μm.

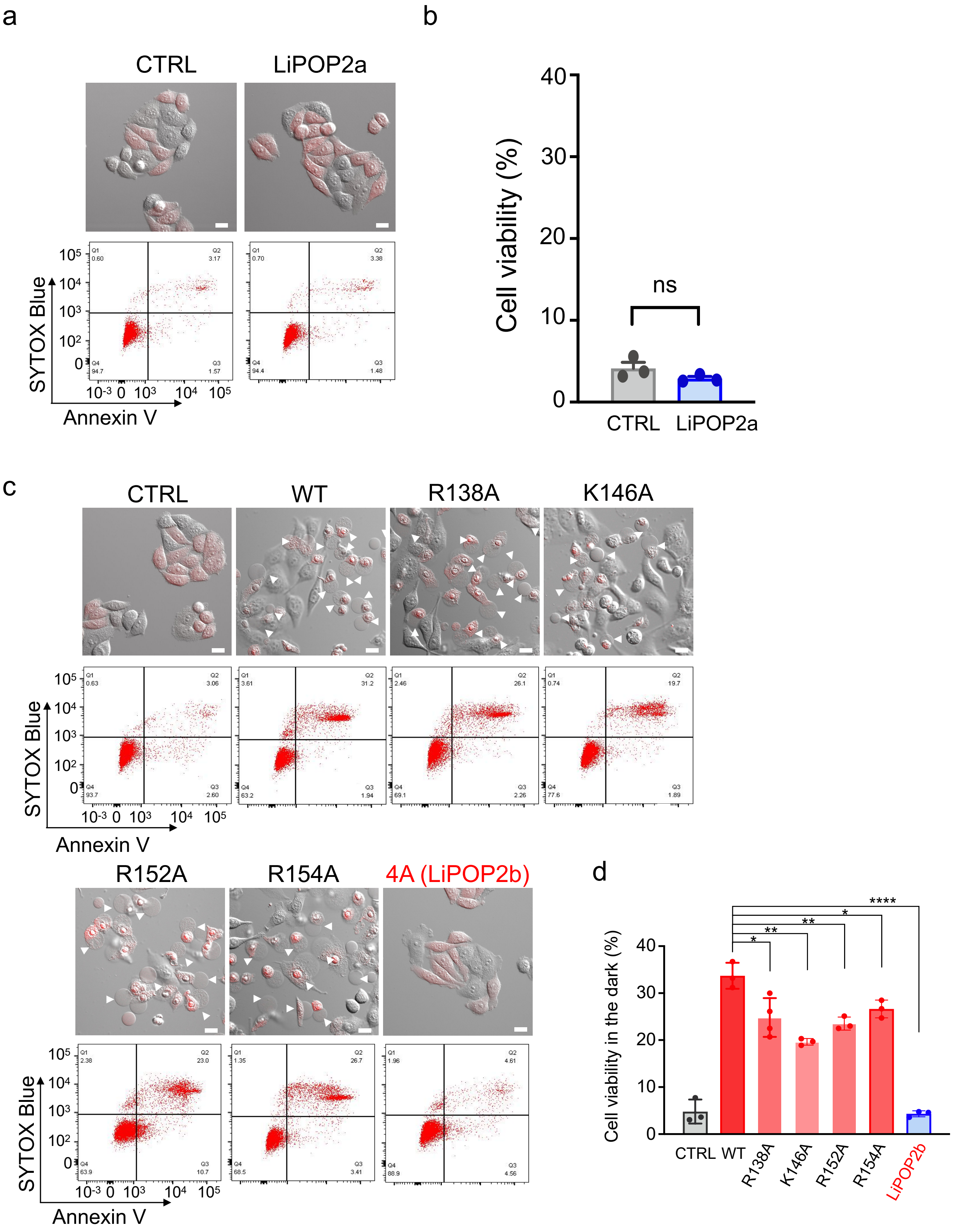

a
CTRL
LiPOP2a
SYTOX Blue
Annexin V
Q1 0.60
Q2 3.17
Q4 94.7
Q3 1.57
Q1 0.70
Q2 3.38
Q4 94.4
Q3 1.48
b
Cell viability (%)
ns
CTRL
LiPOP2a
c
CTRL
WT
R138A
K146A
Q1 0.63
Q2 3.06
Q4 93.7
Q3 2.60
Q1 3.61
Q2 31.2
Q4 63.2
Q3 1.94
Q1 2.46
Q2 26.1
Q4 69.1
Q3 2.26
Q1 0.74
Q2 19.7
Q4 77.6
Q3 1.89
R152A
R154A
4A (LiPOP2b)
Q1 2.38
Q2 23.0
Q4 63.9
Q3 10.7
Q1 1.35
Q2 26.7
Q4 68.5
Q3 3.41
Q1 1.96
Q2 4.61
Q4 88.9
Q3 4.56
d
Cell viability in the dark (%)
****
*
**
**
*
CTRL
WT
R138A
K146A
R152A
R154A
LiPOP2b

Figure S5 | Optical control pyroptosis. (Related to **Fig. 4**)

Data were shown as mean ± s.d. from three independent assays. Scale bar, 10 μm. ns, not significant; * $P < 0.05$; ** $P < 0.01$; **** $P < 0.0001$ when compared to the corresponding control group (two-tailed Student's *t*-test).

a, (Top) Confocal images of HeLa cells transiently transfected with LiPOP2a or the corresponding control (LOV2-P2A-Zdk-mCh-GSDMD-CT) without light stimulation. (Bottom) Cell viability was analyzed by flow cytometry using Annexin V and SYTOX blue staining.

b, Quantification of the percentage of dead HeLa cells expressing LiPOP2a marked by both Annexin V and SYTOX blue staining prior to photostimulation.

c, (Top) Confocal images of HeLa cells transiently expressing LiPOP2b constructs or the control (cpLOV2-mCh) prior to photostimulation. (Bottom) Cell variability was analyzed by flow cytometry using Annexin V and SYTOX Blue staining (lower panel). White arrowheads indicated pyroptotic cells.

d, Quantification of double positive HeLa cells expressing the indicated constructs in the dark (as shown in **c**).

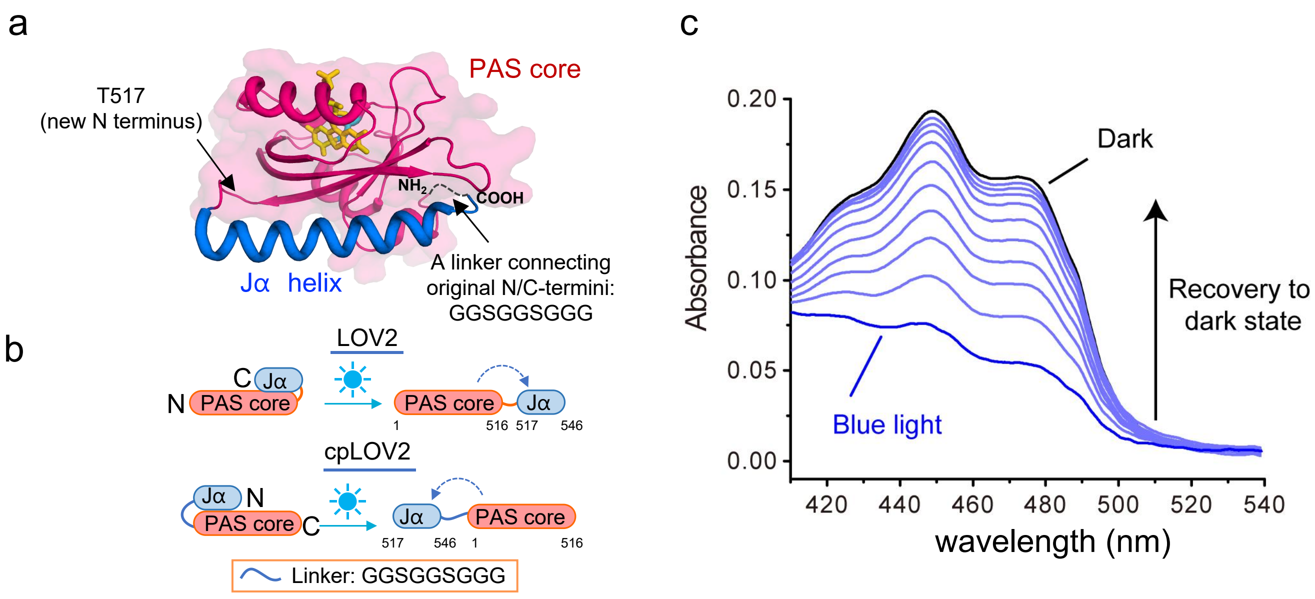

Figure S6 | LOV2 retains photosensitivity after circular permutation. (Related to **Fig. 4**)

a, The 3D structure of LOV2 (PDB entry: 2V0W). The original N/C-termini of AsLOV2 were covalently connected by a linker, GGSGGSGGG via circular permutation, with concomitant generation of new termini between residues G516 and T517. The light-absorbing cofactor flavin mononucleotide (FMN) and the nearby cysteine residue (C450) were highlighted in yellow and cyan, respectively. PAS, Per-Arnt-Sim domain (magenta); Jα , the C-terminal helix undergoing light-dependent unfolding (blue).

b, Cartoon showing the domain organizations of WT LOV2 and circularly permuted LOV2 (cpLOV2).

c, UV-Vis spectra showing the absorbance changes of cpLOV2 when recovering from the lit state (blue) to the dark state (black). Signals were recorded every 25 s. This result indicated that circular permutation did not disrupt the photochemical property of cpLOV2.

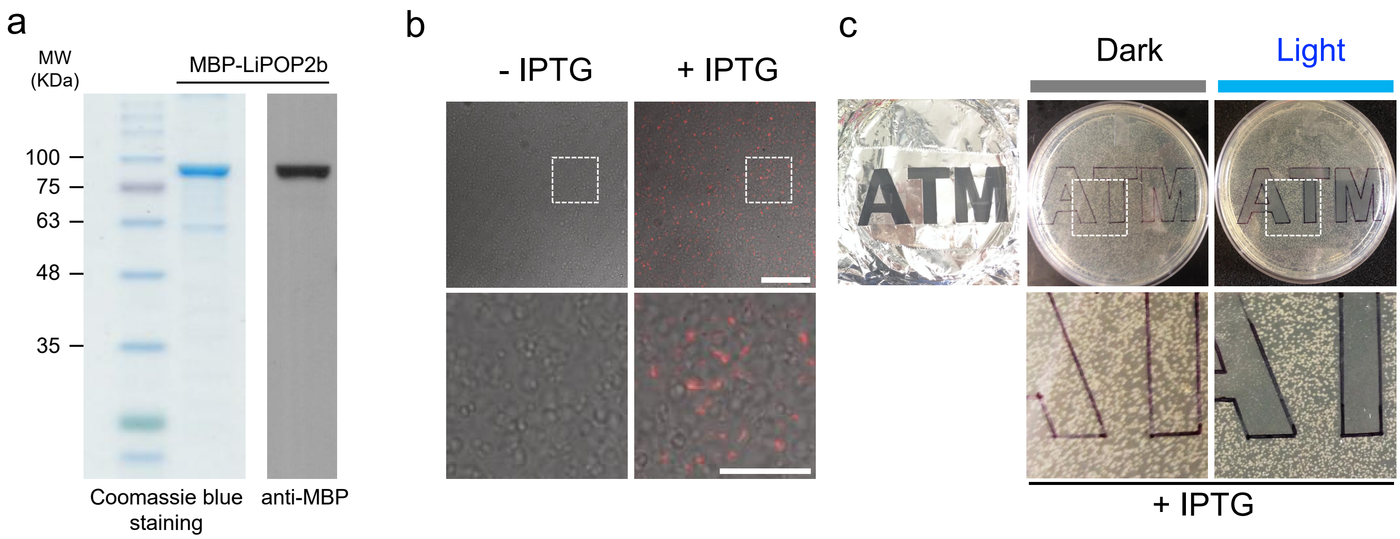

Figure S7 | Expression and purification of LiPOP2b in *E.coli.* (Related to **Fig. 5**)

Photostimulation was applied at 470 nm with a power density of 4 mW/cm^2.

a, SDS-PAGE showing the recombinant MBP taged LiPOP2b protein purified from BL21 *E.coli* cells. (left) Coomassie blue staining; (right) immunoblotting with an anti-MBP antibody.

b, Confocal images of *E.coli* cells expressing LiPOP2b (mCh-positive). Transformed BL21 *E.coli* cells were spread on LB plates with or without 0.5 mM IPTG. Scale bar, 10 µm. High-magnification images of boxed areas are shown to the bottom (Scale bar, 5 µm).

c, The plasmid encoding LiPOP2b was transformed into the BL21 *E.coli* cells. Cells were spread on LB plates with 0.5 mM IPTG to induce LiPOP2b expression. The plates were covered with aluminum foil but with a selected region (marked by the symbol "ATM") exposed to pulsed blue light for 16 h (10 s ON + 50 s OFF) at 37 ^{o}C.

Table S1. Complete blood routine analysis results of mice injected with PBS or Fluorofurimazine (FFz).

Parameters	Normal range	PBS	FFz
WBC (K/μL)	1.8-10.7	2.1	1.91
LY (K/μL)	0.9-9.3	1.61	1.68
MO (K/μL)	0.0-0.4	0.22	0.06
NE (K/μL)	0.1-2.4	0.27	0.16
RBC (M/μL)	6.36-9.42	9.27	8.89
HGB (g/dL)	11.0-15.1	14.8	14.9
HCT (%)	35.1-45.4	38.26	39.63
MCV (fL)	45.4-60.3	60	60
MCH (pg)	14.1-19.3	15.3	15.1
MCHC (K/μL)	30.2-34.2	25.3	25
RDW (K/μL)	12.4-27.0	22.3	23.1
MPV (fL)	5.0-20.0	8.2	9.2

Abbreviations: White blood cell (WBC), Neutrophils (NE), Lymphocytes (LY), Monocytes (MO), Eosinophils (EO), Basophils (BA), Red blood cell (RBC), Hemoglobin (HGB), Hematocrit (HCT), Mean corpuscular volume (MCV), mean corpuscular hemoglobin (MCH), Mean corpuscular hemoglobin concentration (MCHC), Red blood cell distribution width (RDW), Mean platelet volume (MPV).

Table S2. Summary of activation kinetics of designed optogenetic devices.

<table>
<tr><th rowspan="2">Optogenetic Tools</th><th rowspan="2">Stimulation</th><th colspan="2">Half-lives ($t_{1/2}$)</th><th rowspan="2" colspan="2">Time to reach 50% of PM perforation</th></tr>
<tr><td>RIPK1/3 clustering</td><td>Cytosol-to-PM Translocation</td></tr>
<tr><td>mCherry-CRY2-RIPK1</td><td>Blue light (470-490 nm, 4 mW/cm^2)</td><td>3.6 ± 0.7 min</td><td>N/A</td><td colspan="2">N/A</td></tr>
<tr><td>mCherry-CRY2-RIPK3</td><td>Blue light (470-490 nm, 4 mW/cm^2)</td><td>39.1 ± 0.2 min</td><td>N/A</td><td colspan="2">N/A</td></tr>
<tr><td>LiPOP1</td><td>Blue light (470-490 nm, 4 mW/cm^2)</td><td>N/A</td><td>12.8 ± 1.1 s</td><td colspan="2">18.0 ± 4.0 min</td></tr>
<tr><td>LiPOP1-NanoLuc</td><td>Fz or FFz (10 μM)</td><td>N/A</td><td>46.1 ± 17.3 s</td><td colspan="2">< 30 min</td></tr>
<tr><td rowspan="2">LiPOP2a</td><td rowspan="2">Blue light (470-490 nm, 4 mW/cm^2)</td><td rowspan="2">N/A</td><td rowspan="2">N/A</td><td>HeLa</td><td>6.1 ± 0.4 h</td></tr>
<tr><td>Engineered T-cells</td><td>4.0 ± 0.3 h</td></tr>
<tr><td rowspan="2">LiPOP2b</td><td rowspan="2">Blue light (470-490 nm, 4 mW/cm^2)</td><td rowspan="2">N/A</td><td rowspan="2">N/A</td><td>HeLa</td><td>6.2 ± 0.1 h</td></tr>
<tr><td>Engineered T-cells</td><td>3.0 ± 0.3 h</td></tr>
</table>

Table S3. Side-by-side comparisons of representative molecular tools designed to induce cell death with light.

Tools	Stimulation	Cell types	Photoreceptors /Photosensitizers	Toxic modules /molecules	Mechanism(s)	Efficiency	References
LiPOP1 / LiPOP1-NanoLuc	Blue light (470-490 nm, 4 mW/cm^2)	HeLa, 786-O, B16, xenograft mouse models of tumor	CRY2	MLKL-NT	Necroptosis	~30 min	This study
LiPOP2	Blue light (470-490 nm, 4 mW/cm^2)	HeLa, Jurkat CAR T-cells, bacteria	LOV2/Zdk and cpLOV2	GSDMD-NT	Pyroptosis	~3-6 h	This study
KillerRed	Green light (540-580 mm)	HEK293T, B16, bacteria, zebrafish	KillerRed	ROS	Mitochondrial targeting, apoptosis; PM targeting; lipid oxidation (10-30 min)	Cytosolic distribution; 10 min ~40-60% cell death; Mitochondrial localization, 45 min for all the cells death	[1] [2] [3]
SuperNovaGreen	Blue light (440-480 nm)	HeLa cells	KillerRed - V44A	ROS	Mitochondrial matrix; PM perturbance	7 hours to kill 40% cells	[4]
miniSOG	Blue light (455-495 nm)	*C. elegans*	miniSOG	ROS	Mitochondrial targeting; apoptosis	Behavioral defects within 30 min	[5], [6]

Abbreviations: ROS, reactive oxygen species; V, valine; A, alanine.

Table S4. Comparison of optogenetic stimulation approaches with chemogenetics.

Approaches	Stimulus	Tissue penetration	Temporal control	Spatial control	Other notes
Conventional optogenetics	Blue or green light (450-500 nm)	< 1 mm	Yes; ms to seconds; fully reversible	Yes; subcellular precision	Not ideal for non-invasive *in vivo* studies; Long-term stimulation is toxic
NanoLOGS	Fz or FFz	Any tissues	Yes; seconds; reversible	Poor or partial control if combined with tissue /cell-specific expression	FFz is a safer and more potent substrate suitable for *in vivo* optogenetic studies
UCNPs	NIR (980 nm)	Up to 2-3 cm	Yes; seconds; fully reversible	Yes; superior resolution	Long-term stimulation will generate local heating
Chemogenetics	Chemicals	Any tissues	Yes; partially reversible or irreversible	Poor	Potential side effects from the chemicals

Supplementary References

[1] M. E. Bulina, K. A. Lukyanov, O. V. Britanova, D. Onichtchouk, S. Lukyanov, D. M. Chudakov, *Nat Protoc* **2006**, *1*, 947-953.

[2] M. E. Bulina, D. M. Chudakov, O. V. Britanova, Y. G. Yanushevich, D. B. Staroverov, T. V. Chepurnykh, E. M. Merzlyak, M. A. Shkrob, S. Lukyanov, K. A. Lukyanov, *Nat Biotechnol* **2006**, *24*, 95-99.

[3] C. Teh, D. M. Chudakov, K. L. Poon, I. Z. Mamedov, J. Y. Sek, K. Shidlovsky, S. Lukyanov, V. Korzh, *BMC Dev Biol* **2010**, *10*, 110.

[4] Y. D. Riani, T. Matsuda, K. Takemoto, T. Nagai, *BMC Biol* **2018**, *16*, 50.

[5] S. Xu, A. D. Chisholm, *Sci Rep* **2016**, *6*, 21271.

[6] Y. B. Qi, E. J. Garren, X. Shu, R. Y. Tsien, Y. Jin, *Proc Natl Acad Sci U S A* **2012**, *109*, 7499-7504.

Captions for Supplementary Movies

Movie S1 | Time-lapse imaging of HeLa cells co-expressing mCh-CRY2-RIPK1 (upper panel; red) and RIPK3-GFP cells (green) or GFP (as control; lower panel; green). Cells were exposed to pulsed stimulation of 488-nm confocal laser (1 s ON for each 5 s). White arrowheads (upper panel) indicated a cell without mCh-CRY2-RIPK1 expression, in which RIPK3-GFP alone did not form puncta. Scale bar, 10 μm.

Movie S2 | Time-lapse imaging of HeLa cells co-expressing mCh-CRY2-RIPK3 (upper panel; red) and MLKL-Venus (green) or Venus (as control; lower panel; Green). Cells were stimulated with pulsed 488-nm confocal laser (1 s ON for each 5 s) for 1 hour. Scale bar, 10 μm.

Movie S3 | Time-lapse confocal imaging of HeLa cells expressing LiPOP1 (red) upon exposure to pulsed 488-nm confocal laser stimulation (1 s ON for each 5 s) for 1 hour. Pacific Blue Annexin V (blue) was used as an indicator for cell death.

Movie S4 | Light-inducible Ca^{2+} influx monitored in LiPOP1 (red)-expressing HeLa cells. Cells were transiently transfected with GCaMP6s-CAAX (green) and exposed to pulsed 488-nm confocal laser stimulation for 30 min (1 s ON for every 30 s). Scale bar, 10 μm.

Movie S5 | Time-lase confocal imaging of 786-O tumor cells with stable expression of LiPOP1-NanoLuc upon treatment with 10 μM Fz for 30 min. The images were captured every 2 seconds. Scale bar, 10 μm.

Movie S6 | Time-lapse confocal imaging of HeLa cells stably expressing LOV2-P2A-Zdk-mCh-GSDMD-CT (as control; left) or LiPOP2a (right) following external blue light stimulation (470 nm, 4 mW/cm^2; pulse of 5 s ON + 30 OFF for 8.5 hours). Scale bar, 10 μm.

Movie S7 | Time-lapse imaging of HeLa cells stably expressing cpLOV2-mCherry (as control; left) or LiPOP2b (right) following exposure to an external blue light (pulse of 5 s ON + 30 OFF for 8.5 hours; 470 nm, 4 mW/cm^2). Scale bar, 10 μm.

Movie S8 | Time-lapse confocal imaging of engineered T cells stably expressing the control (LOV2-P2A-Zdk-mCh-GSDMD-CT; left) or LiPOP2a (right) upon 6 hours of external blue light stimulation (pulse of 5 s ON + 30 OFF, 470 nm, 4 mW/cm^2). Scale bar, 10 μm.

Movie S9 | Live confocal imaging of engineered T cells stably expressing the control (cpLOV2-mCherry; left) and LiPOP2b (right) in response to external blue light illumination (470 nm, 4 mW/cm^2, pulse of 5 s ON + 30 OFF for 6 hours). Scale bar, 10 μm.
